# Health and economic impact of oral PrEP provision across subgroups in western Kenya: a modelling analysis

**DOI:** 10.1136/bmjgh-2024-015835

**Published:** 2025-01-11

**Authors:** Rachel Wittenauer, Linxuan Wu, Sarah Cox, Brian Pfau, Monisha Sharma

**Affiliations:** 1 Department of Pharmacy, University of Washington, Seattle, Washington, USA; 2 Department of Epidemiology, University of Washington School of Public Health, Seattle, Washington, USA; 3 Department of Global Health, University of Washington, Seattle, Washington, USA

**Keywords:** HIV, Kenya, Health economics, Decision Making, Prevention strategies

## Abstract

**ABSTRACT:**

**Introduction:**

Oral pre-exposure prophylaxis (PrEP) is a priority intervention for scale-up in countries with high HIV prevalence. Policymakers must decide how to optimise PrEP allocation to maximise health benefits within limited budgets. We assessed the health and economic impact of PrEP scale-up among different subgroups and regions in western Kenya.

**Methods:**

We adapted an agent-based network model, EMOD-HIV, to simulate PrEP uptake in six counties of western Kenya across seven subgroups including serodiscordant couples (SDCs), adolescent girls and young women (AGYW), adolescent boys and young men, women with multiple partners and men with multiple partners. We modelled 5 years of PrEP provision assuming 90% PrEP uptake in the prioritised subgroups and evaluated outcomes over 20 years compared with a no PrEP scenario. All results are presented in 2021 USD$.

**Results:**

Population PrEP coverage was highest in the broad AGYW scenario (8.3%, ~2 fold higher than the next highest coverage scenario) and lowest in the SDC scenario (0.37%). Across scenarios, PrEP averted 4.5%–21.3% of infections over the 5-year implementation. PrEP provision to SDCs was associated with the lowest incremental cost-effectiveness ratio (ICER), $245 per disability-adjusted life year (DALY) averted (CI $179 to $435), followed by women and men with multiple partners ($1898 (CI $1002 to $6771) and $2351 (CI $1 831 to $3494) per DALY averted, respectively). Targeted strategies were more efficient than broad provision even in high HIV prevalence counties; PrEP scale-up for AGYW with multiple partners had an ICER per DALY averted of $4745 (CI $2059 to $22 515) compared with $12 351 for broad AGYW (CI $7 050 to $33,955). In general, ICERs were lower in counties with higher HIV prevalence.

**Conclusions:**

PrEP scale-up can avert substantial HIV infections and increasing PrEP demand for subgroups at higher risk can increase efficiency of PrEP programmes. Our results on health and cost impact of PrEP across geographic regions in western Kenya can be used for budgetary planning and priority setting.

**WHAT IS ALREADY KNOWN ON THIS TOPIC**

- Pre-exposure prophylaxis (PrEP) for HIV prevention is an effective strategy to reduce HIV incidence and is shown to be cost-effective in narrowly defined, high-risk populations in Eastern and Southern Africa (ESA), but evidence is lacking on relative efficiency of broadening PrEP access to larger population subgroups.

**WHAT THIS STUDY ADDS**

- In this agent-based model across six counties in western Kenya, we found that targeted strategies are more cost-effective than broad PrEP provision. In addition to serodiscordant couples and adolescent girls and young women (who are often prioritised in HIV prevention programmes), we found that PrEP uptake by men and women aged 25–49 with multiple partners is also relatively efficient for reducing population-level HIV incidence.

**HOW THIS STUDY MIGHT AFFECT RESEARCH, PRACTICE OR POLICY**

- In the context of shrinking donor funding, findings may help inform demand-generation efforts and priority-setting for national HIV prevention programmes in ESA.

## Introduction

 Despite strides in treatment and prevention, HIV remains a leading cause of morbidity and mortality in Eastern and Southern Africa (ESA),^1 2^ placing considerable economic strain on households and health systems.^3^ Expansion of prevention is needed to achieve global targets of 90% reduction in new HIV infections by 2030.^3 4^ One effective tool in the HIV prevention portfolio is daily oral pre-exposure prophylaxis (PrEP), which reduces HIV acquisition by over 90% when used with high adherence.^5^ PrEP is safe, does not require consent from sexual partners and does not involve a cold chain, which positions it to be well suited for widespread use among those with HIV risk indication.

Many countries in ESA, including Kenya, have prioritised PrEP expansion to achieve the ambitious Joint United Nations Programme on HIV/AIDS (UNAIDS) target of 10 million people on PrEP by 2030.^6^ Kenya was the first country in Africa to provide oral PrEP as a national public sector programme after its approval in 2017.^7 8^ The Kenya Ministry of Health (MOH)’s AIDS Strategic Framework 2021–2025^3 9^ outlines guidance and priorities to achieve the country’s HIV reduction goals, for which PrEP is a vital tool. Despite disruptions due to the COVID-19 pandemic, the programme has achieved steady increases in PrEP coverage^10^ —as of August 2022, there were >2 56 000 PrEP initiations in the country.^11^ However, in the era of shrinking donor funding for HIV, policymakers must decide how to prioritise PrEP services to maximise population health benefits within limited budgets. Previous modelling has suggested that PrEP scale-up can be cost-effective when prioritised to certain subgroups, such as female sex workers (FSWs) and individuals with multiple condomless sex partners.^12^,^15^ However, there are little data on the impacts of PrEP provision to other subgroups including adolescents and young adults, and older men and women with sexual behaviour associated with HIV risk. Furthermore, most previous modelling studies have not incorporated the beneficial effects PrEP scale-up has on increasing HIV testing and linkage to care.^16^ Understanding the comparative impact of PrEP uptake across subgroups and geographic regions can inform demand generation and priority- setting strategies to optimise roll-out.

We sought to assess the health and economic impact of PrEP scale-up among different priority groups across six counties in western Kenya with varying HIV prevalence. We used primary data on PrEP provision costs from Kenya and evaluated the incremental cost-effectiveness of each PrEP scale-up scenario relative to a baseline scenario of no PrEP availability. Our findings generate evidence about the relative benefits of prioritising PrEP to different subgroups in a generalised epidemic setting.

## Methods

### Mathematical model

We adopted an agent-based network model, EMOD-HIV for this analysis.^17^,^21^ The model uses monthly time steps and simulates HIV infection dynamics, disease progression, heterosexual relationship patterns and includes an adaptable HIV care cascade including HIV testing and linkage and retention on antiretroviral therapy (ART). The PrEP prevention cascade incorporates uptake, adherence, persistence and re-engagement. Modelled individuals can form long-term or causal partnerships and engage in condomless sex. The model was calibrated to the HIV epidemic in western Kenya using empiric data on HIV prevalence across six geographic counties: Siaya, Kisumu, Nyamira, Kisii, Homa Bay and Migori. We parameterised the model with epidemiological data from western Kenya including fertility, mortality, voluntary male circumcision coverage and estimated number of FSWs and male clients of FSWs in each of the six counties. Model calibration used an optimisation algorithm that identified an optimal set of input parameters given the empiric data. We selected 100 good-fitting parameter sets to conduct the analysis. Model data and calibration targets are found in online supplemental section 1, tables S1–S7 and figures S1–S7.

Model inputs were obtained from published literature, empiric data and expert opinion (table 1 and online supplemental materials section 2). Economic costs were adjusted for inflation to 2021 and converted to USD as needed.^22^ Analyses from the MOH (payer) perspective included direct medical costs of HIV testing, PrEP initiation, HIV-related hospitalisations and ART (table 1 and online supplemental table S8).

**Table 1:** Key model parameters and assumptions

	Estimate(sensitivity range)	References and notes
**Costs**		
Testing and diagnosis		
Facility-based HIV-positive test (background)	$3.68	Meisner *et al*^40^
Facility-based HIV-negative test (background)	$2.64	Meisner *et al*^40^
HIV test unit cost for PrEP initiation (if negative)	$2 ($0.50–$8)	Expert opinion
Referral if HIV test for PrEP initiation is positive	$4 ($1–$8)	Mangale *et al*^41^; Meisner *et al*^40^
Cost of illness and death for PLWH		
Average monthly care costs, CD4 count of>350	$8.59	Eaton *et al*^42^*
Average monthly care costs, CD4 200–350	$30.38	Eaton *et al*^42^*
Average monthly care costs, CD4<200	$110.30	Eaton *et al*^42^*
Costs of death	$105.68	Eaton *et al*^42^*
Costs of medications		
30 days of PrEP drugs	$7.00 ($5–$15)	Wanga *et al*^38^; Roberts *et al*^39^
PrEP initiation visit	$6.50 ($5–$15)	Wanga *et al*^38^; Roberts *et al*^39^
PrEP continuation visit	$5.00 ($3–$10)	Wanga *et al*^38^; Roberts *et al*^39^
Annual ART costs	$140.89	Phillips *et al*^13^; Larson *et al*^43^; The Global Fund 2023. Inclusive of delivery costs, and assumes 3% of patients are given second-line ART^44^
**Model assumptions**		
Analytic time horizon	2022–2042	Assumption
PrEP implementation period	2022–2027	Assumption
PrEP effectiveness	75%	Phillips *et al*^13^; Baeten *et al*^26^
PrEP continuation if eligible	75%	Assumption
Disability-adjusted life year (DALY) weights		
HIV-positive and on ART	0.078 (95% CI: 0.052 to 0.111)	Global Burden of Disease Study^2^
HIV+ (CD4>350)	0.274 (95% CI: 0.184 to 0.377)	Global Burden of Disease Study^2^
HIV+ (CD4 200–350)	0.312 (95% CI: 0.217 to 0.418)	Global Burden of Disease Study^2^
HIV+ (CD4<200)	0.642 (95% CI: 0.470 to 0.792)	Global Burden of Disease Study^2^

All costs are presented in 2021 USD

* Adjusted for inflation and GDP per capita ratio.

ARTantiretroviral therapyGDPGross Domestic ProductPLWHPersons living with HIV/AIDSPrEPpre-exposure prophylaxis

### Modelled scenarios

We evaluated the health and economic impact of PrEP provision separately among seven subgroups based on those listed in the Kenyan MOH’s strategic plan as well as input from in-country experts^3 9^: (1) individuals aware that their partner is diagnosed with HIV but is not on ART (henceforth defined as serodiscordant couples (SDCs)), (2) adolescent girls and young women (AGYW) aged 15–24 with multiple concurrent partners, (3) all AGYW, (4) women aged 25–49 with multiple partners in the past 3 months, (5) adolescent boys and young men (ABYM) with multiple concurrent partners, (6) all ABYM and (7) men aged 25–49 with multiple partners in the past 3 months (online supplemental table S9). Across scenarios, additional PrEP eligibility criteria included having at least one partner and not being known to have HIV. Each PrEP scenario was compared with a counterfactual of no PrEP availability.

Across intervention scenarios, PrEP was allocated to 90% of eligible individuals. Prior to PrEP initiation, eligible modelled persons underwent HIV testing to verify negative status; those testing HIV-positive were assumed to link to ART at background rates. Individuals who tested HIV-negative received a 1-month supply of PrEP and individuals that remained eligible had a 75% probability of returning for a 3-month PrEP refill (and 75% probability returning for a refill every 3 months thereafter). Continuation probabilities were found in prior analyses to result in mean duration of 3 months of PrEP use, consistent with real-world data.^523^,^25^ PrEP discontinuation occurred due to loss-to-follow-up or if eligibility criteria were no longer met. Individuals who discontinued PrEP could reinitiate PrEP at the same rate if they met eligibility criteria.^21^ Individuals underwent HIV testing at PrEP initiation and each refill visit, with HIV test sensitivity varying by time since HIV infection (online supplemental section 2.1, tables S10–S11 and figure S3). We assumed PrEP effectiveness of 75% based on empiric data accounting for observed adherence.^26^ We used PrEP uptake, persistence and effectiveness probabilities that were higher than those reported in the literature to evaluate the upper bound impact and relative efficiency of PrEP use across subgroups assuming similar uptake. We used a 20-year time horizon (2022–2042) and assumed PrEP was implemented for the first 5 years starting 2022 and ending in 2027. The 5-year implementation period aligns with the typical timeframe of MOH planning activities, and the 20-year analytic time horizon enabled us to capture long-term outcomes associated with PrEP provision.

### Analysis

For each scenario, we assessed number of incident HIV infections, HIV-related deaths and costs over 5 years of PrEP implementation and the 20-year time horizon. We calculated percentage of HIV infections, HIV-related deaths and disability-adjusted life years (DALYs) averted compared with a scenario of no PrEP availability. We also calculated the counterfactual HIV incidence among each of the prioritised subgroup assuming PrEP had not been available. We conducted a cost-effectiveness analysis following the Consolidated Health Economic Evaluation Reporting Standards (CHEERS) guidelines to evaluate relative efficiency of PrEP provision among subgroups.^27^ We calculated the incremental cost-effectiveness ratio (ICER) as the additional cost divided by the additional health benefit (in DALYs) of each scenario compared with the counterfactual of no PrEP utilising the MOH (payer) perspective. Following published guidelines, we applied a 3% discount rate to both costs and DALYs.^28^ To evaluate the impact of geographic heterogeneity and variations in HIV prevalence, we assessed health and economic outcomes separately for all six counties in western Kenya. HIV prevalence ranged from 3% in Kisii to 20% in Homa Bay. For each outcome, we calculated 90% credible intervals (CIs) across the 100 parameter sets to assess parameter uncertainty. Analysis of model outputs was conducted in R V.4.0.3.^29^

### Sensitivity analyses

We conducted one-way sensitivity analyses to assess the robustness of our findings to model inputs. We varied costs, DALY weights, PrEP uptake, PrEP effectiveness and persistence (table 1). While our base case scenarios used costs of facility-based PrEP delivery and provider-administered HIV testing, costs used in sensitivity analyses reflected delivery costs associated with community-based channels such as pharmacies and mobile clinics as well as HIV self-testing for PrEP provision.^30^ We report findings of one-way sensitivity analyses for one illustrative scenario, high-risk AGYW, as the relative impacts of our assumptions on the ICERs are likely similar across scenarios.

We also assessed the impact of lower PrEP uptake among each subgroup (50% vs 90% in the base case). Additionally, we conducted the cost-effectiveness analysis from the societal perspective and included participant costs (eg, costs of informal medical care, costs of transportation to receive PrEP, travel time (in minutes, prorated using annual average income) and lost wages due to missing work from being ill with HIV) and opportunity costs (eg, lost gross domestic product due to absenteeism, lost productivity due to premature mortality from HIV).

### Patient and public involvement

Patients were not directly involved in forming this research question, designing this study or conducting this study. However, the subject of this analysis—modelling expanded use of PrEP to broad populations—draws from heightened calls from patients and patient advocacy groups globally who place pressure on governments, manufacturers and funders to improve the accessibility of HIV prevention and treatment options, including PrEP, to all people who can benefit from them.

## Results

### Health impact

**Figure 1:**
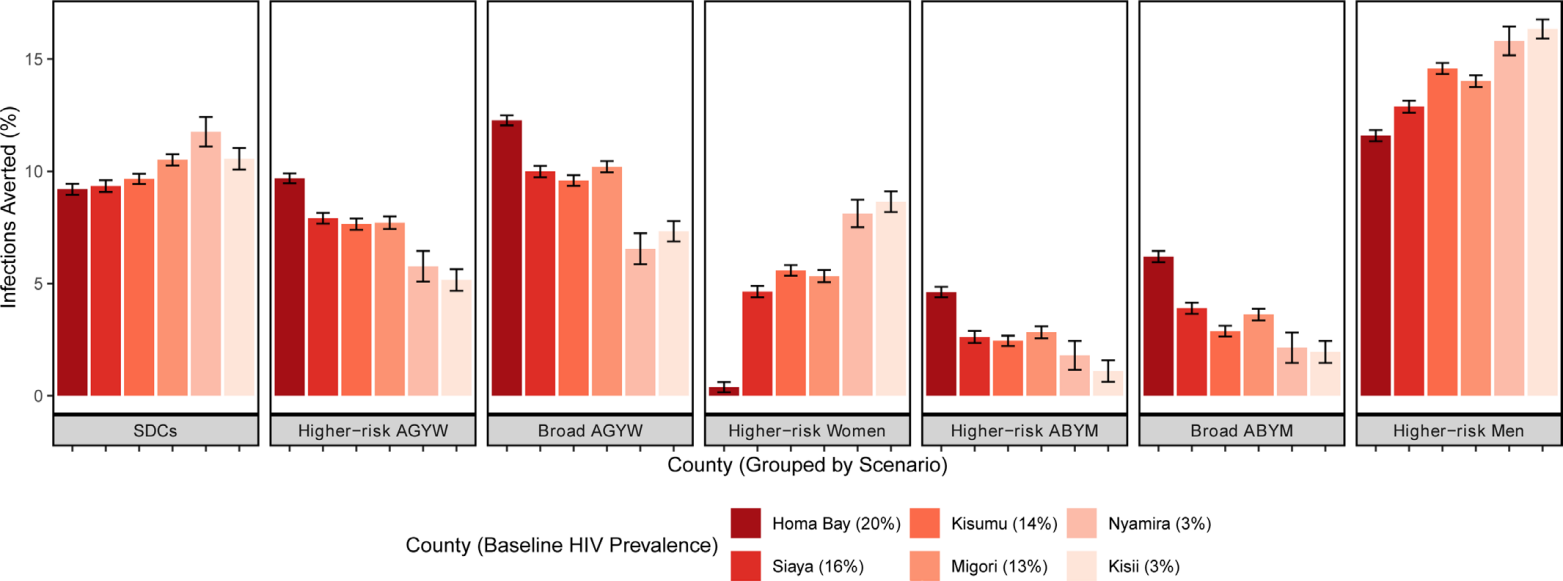
Infections averted by scenario and county. ABYM, adolescent boys and young men; AGYW, adolescent girls and young women; SDCs, serodiscordant couples.

**Figure 2:**
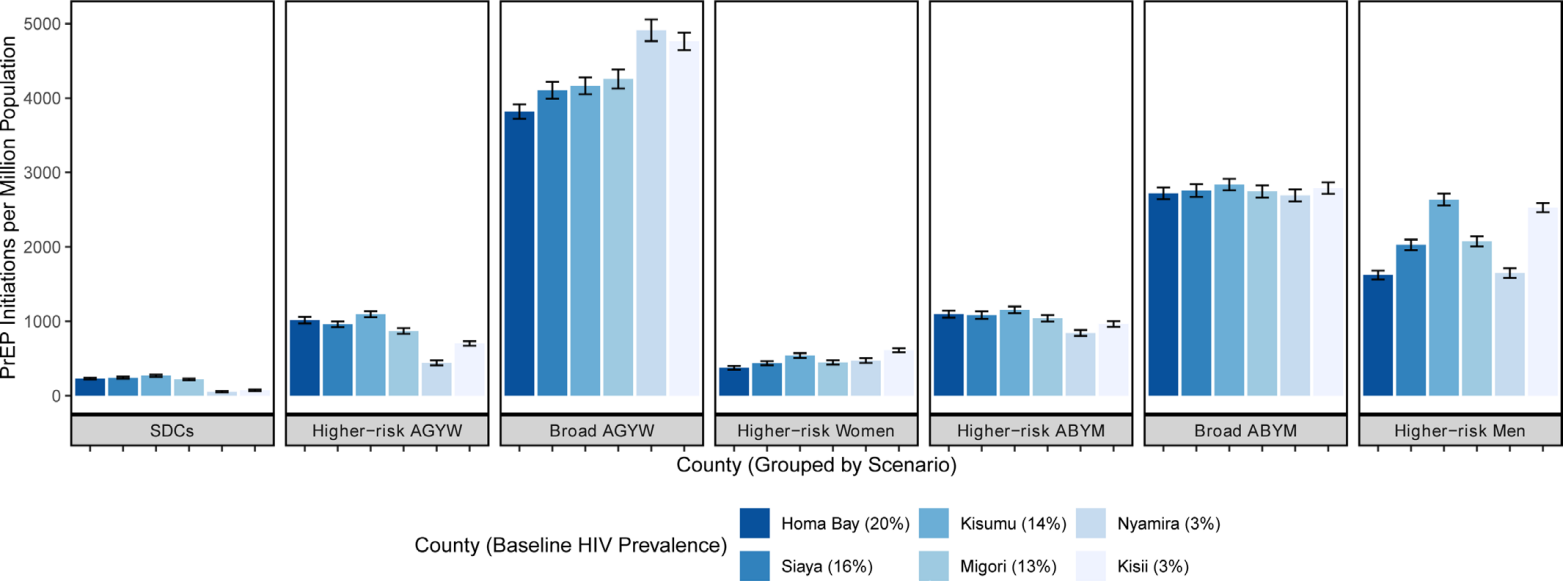
PrEP initiations per million population by scenario and county. ABYM, adolescent boys and young men; AGYW, adolescent girls and young women; PrEP, pre-exposure prophylaxis; SDCs, serodiscordant couples.

All PrEP scenarios were projected to avert HIV incidence and mortality (table 2 and figure 1). Population PrEP coverage over 5 years was highest in the broad AGYW scenario (8.3%), approximately two times that of the next highest coverage scenario (4.7% for broad ABYM); the lowest PrEP coverage scenario was SDCs (0.37%), reflecting the size of the subgroups. HIV incidence reduction was highest in scenarios of PrEP provision to men aged 25–49 years with multiple partners in the past 3 months (21.6%) and broad AGYW (21.3%), followed by SDCs (17.6%), and higher risk AGYWs (14.9%). The smallest HIV incidence reductions were observed in scenarios of provision to broad ABYM (6.7%), followed by higher risk ABYM (4.5%). PrEP coverage and health impact varied by geographic location due to differences in age structure, HIV prevalence and size of target populations, but the rank order of most effective scenarios remained similar to that of western Kenya overall (table 1, figures1^3^). In Homa Bay, the region with the highest HIV prevalence (20%), the relative proportion of HIV incidence reduction from prioritising PrEP for higher risk women was smaller than that of lower prevalence regions, for example, Kisii (HIV prevalence, 3%): incidence reductions were 3.8% vs 8.7% in Homa Bay and Kisii, respectively. However, the absolute number of HIV infections averted was higher in Homa Bay due to the higher prevalence, which translates to higher absolute numbers of people with HIV; this pattern was also observed in higher risk men. In contrast, broader PrEP provision averted a relatively higher proportion of HIV infections in Homa Bay versus Kisii. For example, PrEP provision to broad AGYW averted 12.3% of HIV infections in Homa Bay and 7.3% in Kisii; similar patterns were observed in ABYM and higher risk AGYW. Across counties, the number of PrEP initiations was substantially higher in the broad AGYW scenario than in all other scenarios (figure 2, online supplemental figure S4). Additional health impact results are found in online supplemental Section 3.1, table S12, figure S5.

**Table 2:** PrEP outcomes by scenario in western Kenya (median, 90% CI)*†

	Counterfactual HIV incidence in priority subgroup with no PrEP availability‡ (2022–2027)	Population PrEP coverage(age 15–65)(2022–2027)	Infections averted(2022–2027)	Deaths averted(2022–2042)	DALYs averted(2022–2042)	Incremental costs($ millions)(2022–2042)	ICER§(2022–2042)
Baseline	--	0%	(ref)	(ref)	(ref)	(ref)	(ref)
SDCs	Men: 4.338 (0.822)Women: 10.634 (0.636)	0.37%(0.34–0.4%)	17.61%(15.58–20.07%)	4.13%(2.83–5.49%)	32 517(17 668–46 856)	$8($7–9)	$245($179–$435)
Higher-risk AGYW	1.151 (0.102)	2.03%(1.94–2.13%)	14.93%(12.17–16.95%)	0.93%(−0.42–2.5%)	12 069(−627–29 211)	$61(58–64)	$4745($2059–$22 515)
Broad AGYW	0.472 (0.035)	8.28%(8.11–8.53%)	21.29%(19.21–23.59%)	1.26%(−0.24–2.79%)	20 501(6907–36 625)	$256($250–263)	$12 351($7050–$33 955)
Higher-risk women	1.751 (0.132)	1.06%(0.96–1.12%)	10.3%(7.99–13.05%)	1.67%(0.28–3.23%)	16 090(−850–29 976)	$31($28–33)	$1898($1002– $6771)
Higher-risk ABYM	0.287 (0.033)	1.85%(1.73–1.97%)	4.52%(2.33–7.17%)	0.32%(−1.04–1.51%)	5539(-8099–21 333)	$57($53–60)	$6622($2447–$35 253)
Broad ABYM	0.184 (0.018)	4.71%(4.5–4.87%)	6.68%(4.05–8.44%)	0.54%(−0.74–1.77%)	6055(−7525–22 622)	$147($140–151)	$15 945($6358 - $151 949)
Higher-risk men	0.458 (0.032)	4.21%(4.08–4.34%)	21.58%(20.03–24.02%)	6.73%(5.27–8.04%)	53 026(35 331 – 67 706)	$124($120–128)	$2351($1831–$3494)

* Health impacts are compared tocompared with baseline scenario of no PrEP availability.

† Each scenario is defined as: SDCs) —all individuals aware that their partner is diagnosed with HIV but is not on ART; higher-risk AGYW) —adolescent girls and young women aged 15–24 with multiple concurrent partners; Bbroad AGYW) —all AGYW; Hhigher-risk Wwomen) —women aged 25–49 with multiple partners in the past three3 months, Hhigher-risk ABYM) —adolescent boys and young men with multiple concurrent partners, Bbroad ABYM) —all ABYM, and Hhigher-risk men) —men aged 25–49 with multiple partners in the past 3 months.

‡ Counterfactual HIV incidence per 100 person-years in the scenario’s priority population had there been no PrEP provision; mean (SD).

§ Dominated model runs of each scenario were excluded from ICER calculations: SDCs: 0; higher-risk AGYW: 6; broad AGYW: 1; higher-risk women: 6; higher-risk ABYM: 27; broad ABYM: 18; higher-risk men: 0.

ABYMadolescent boys and young menAGYWadolescent girls and young womenDALYsdisability-adjusted life yearsICERincremental cost-effectiveness ratioPrEPpre-exposure prophylaxisSDCsserodiscordant couples

**Figure 3:**
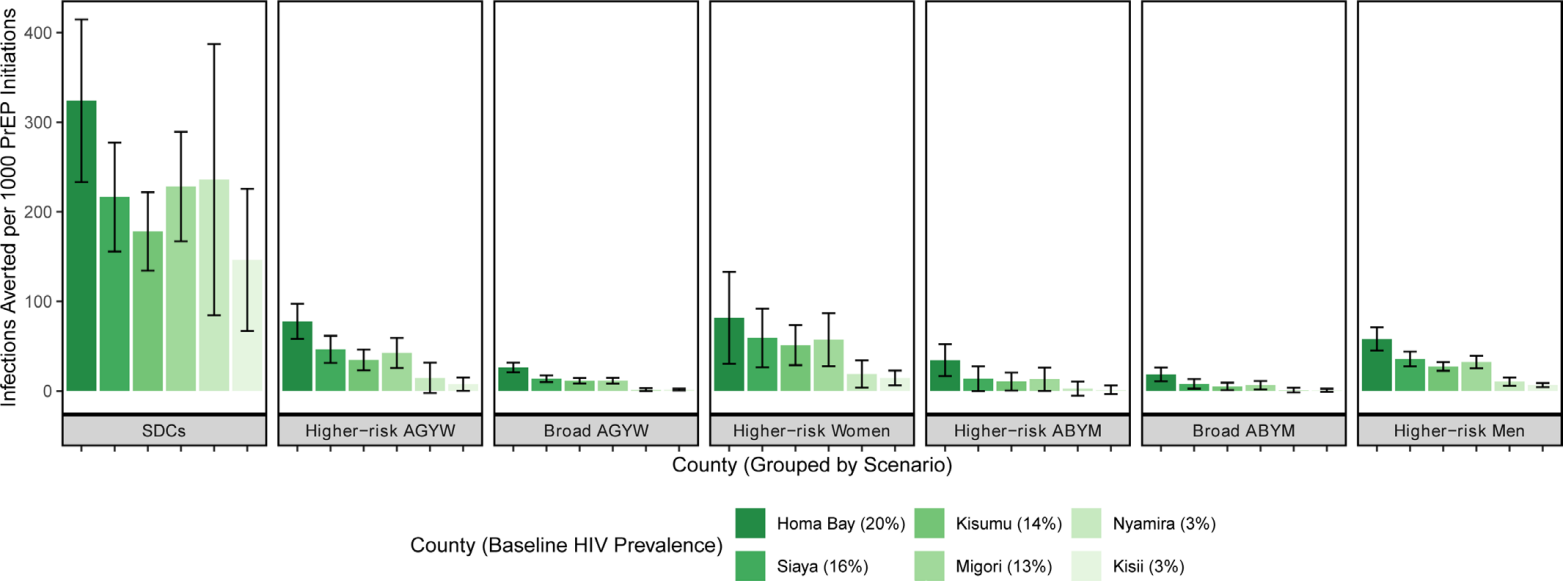
Infections averted per 1000 PrEP initiations by scenario and county. ABYM, adolescent boys and young men; AGYW, adolescent girls and young women; PrEP, pre-exposure prophylaxis; SDCs, serodiscordant couples.

### Economic impact

Assuming 90% uptake among subgroups in western Kenya, PrEP provision to SDCs was associated with the lowest ICER ($245 per DALY averted), followed by women and men with multiple partners ($1898 and $2351 per DALY averted, respectively). Targeted PrEP strategies were more cost-effective than broad strategies; for example, broad AGYW had an ICER of $12 351 per DALY averted compared with higher risk AGYW $4745 per DALY averted. PrEP provision to broad ABYM was associated with the highest ICER, $15 945 per DALY averted. In general, scenarios with higher counterfactual HIV incidence were associated with lower ICERs and those with lower counterfactual incidence had higher ICERs, with the exception of the higher risk men scenario, which had a lower ICER despite lower counterfactual HIV incidence in the subgroup.

The cost-effectiveness of PrEP provision strategies varied by geographic setting and uptake among subgroups; ICERs generally increased with declining HIV prevalence, with the exception of PrEP prioritisation for SDCs, which was associated with low ICERs across counties (table 3a,b). Assuming 90% PrEP uptake, PrEP provision in Homa Bay was associated with ICERs lower than $2000 per DALY averted in all subgroups except broad AGYW and all scenarios of ABYM (table 3a). However, within two counties with the same baseline HIV prevalence of 3% (Nyamira and Kisii), we found differences in cost-effectiveness, with strategies being more cost-effective in Nyamira. Assuming lower PrEP uptake among each subgroup (50% instead of 90%) resulted in fewer HIV infections and deaths averted, but more cost-effective ICERs; the relative ranking of strategies followed the same pattern as the 90% uptake scenarios (table 3b, online supplemental tables S12 and S13, figure S6).

**Table 3:** (a) Median incremental cost-effectiveness ratio for subgroup scenarios by county in Kenya (90% uptake among subgroups) and (b) median incremental cost-effectiveness ratio for subgroup scenarios by county in Kenya (50% uptake among subgroups)*†

County (baseline HIV prevalence)	SDCs	Higher-risk AGYW	Broad AGYW	Higher-risk women	Higher-risk ABYM	Broad ABYM	Higher-risk men
(a)							
Homa Bay (20%)	$142	$1960	$5151	$850	$2827	$5442	$1073
Siaya (16%)	$255	$3377	$8707	$1260	$3170	$7206	$1686
Kisumu (14%)	$317	$3622	$8593	$1274	$3171	$8488	$2011
Migori (13%)	$258	$4038	$10 130	$1059	$2779	$7877	$2082
Nyamira (3%)	$162	$1527	$15 884	$1723	$2353	$7041	$4325
Kisii (3%)	$213	$3971	$18 678	$3347	$3515	$10 637	$7860
(b)							
Homa Bay (20%)	$113	$1378	$3750	$667	$1218	$3330	$778
Siaya (16%)	$152	$1049	$3831	$567	$2545	$2805	$1104
Kisumu (14%)	$163	$1762	$4846	$635	$1586	$4049	$1221
Migori (13%)	$148	$1123	$6931	$557	$1409	$5828	$1243
Nyamira (3%)	$41	$807	$7268	$453	$1216	$4453	$1603
Kisii (3%)	$80	$1461	$9786	$1189	$1561	$4680	$3123

* Green shaded cells indicates lower ICERs (more cost-effective) and brown shaded cells indicates higher ICERs (less cost-effective).

† Each scenario is defined as: SDCs) —all individuals aware that their partner is diagnosed with HIV but is not on ART; higher-risk AGYW) —adolescent girls and young women aged 15–24 with multiple concurrent partners; broad AGYW) —all AGYW; higher-risk women) —women aged 25–49 with multiple partners in the past three3 months, higher-risk ABYM) —adolescent boys and young men with multiple concurrent partners, broad ABYM) —all ABYM, and higher-risk men) —men aged 25–49 with multiple partners in the past 3 months.

ABYMadolescent boys and young menAGYWadolescent girls and young womenARTantiretroviral therapyICERincremental cost-effectiveness ratioSDCsserodiscordant couples

Incremental costs of PrEP scenarios were higher than those of no PrEP availability, with PrEP drugs making up the largest component cost (table 2, online supplemental figure S7a, b). Over a 5-year time horizon, assuming a baseline population size of 3.9 million individuals aged 15–65, PrEP provision to SDCs had the smallest incremental undiscounted cost ($8 million) while PrEP to broad AGYW had the largest costs ($60 million). Costs of HIV-related hospitalisation were lower in all PrEP scenarios compared with the baseline scenario (online supplemental table S14a, b). Additional costing results are found in online supplemental section 3.1, figures S4–S8 and Tables S12–S14.

### Sensitivity analyses

**Figure 4:**
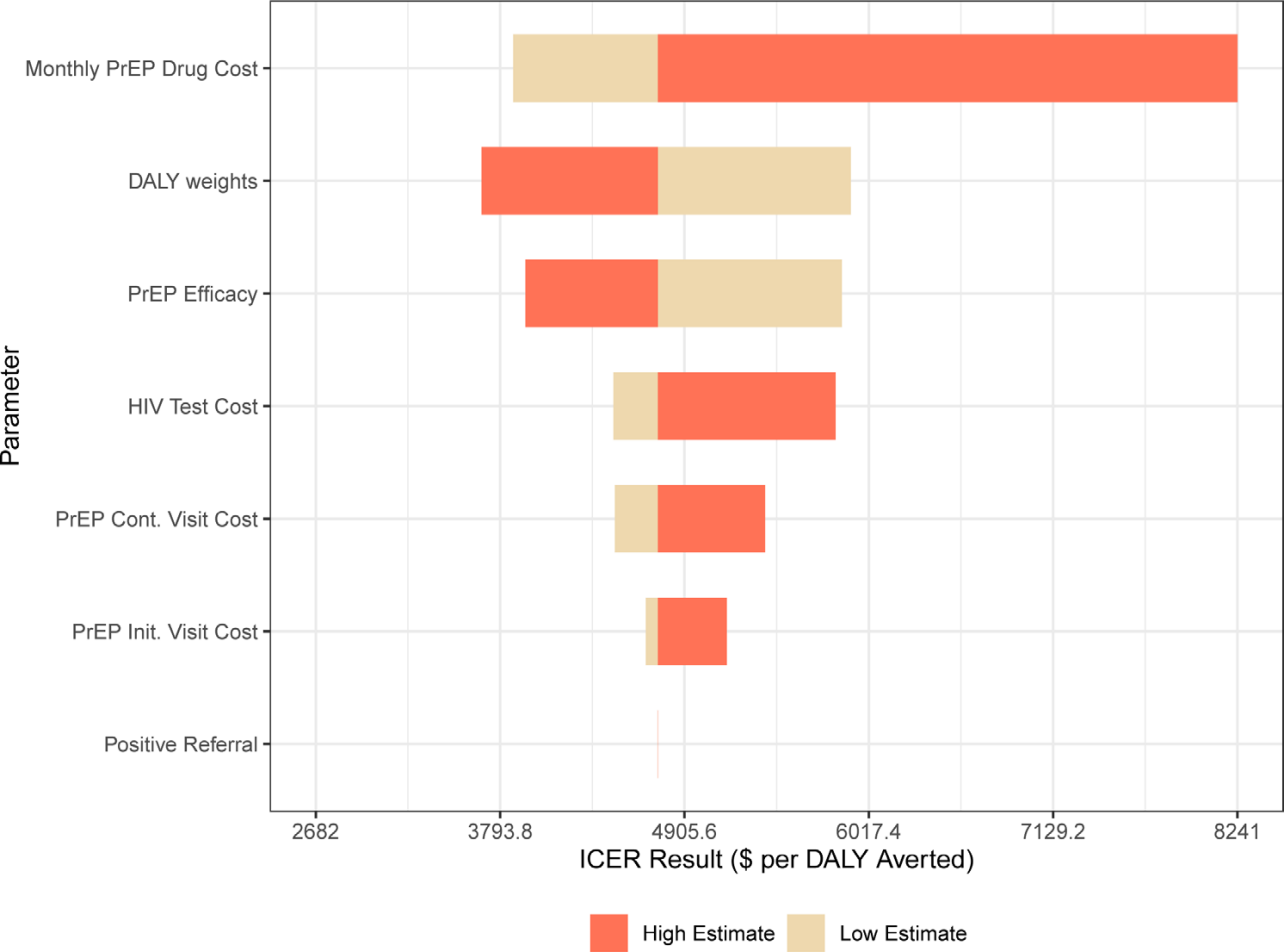
Tornado diagram of sensitivity analyses. DALYs, disability-adjusted life years; ICER, incremental cost-effectiveness ratio; PrEP, pre-exposure prophylaxis.

Results were most sensitive to PrEP drug costs, DALY weights and PrEP effectiveness, while other costs were less influential (figure 4). Utilising the societal perspective for the cost-effectiveness analysis resulted in all scenarios being cost-saving over the 20-year time horizon. Additional results from the sensitivity analyses are found in online supplemental section 3.2, tables S15–S19, figure S9.

## Discussion

We used an established HIV transmission model to evaluate the health impact, cost and relative efficiency of providing PrEP to various subgroups in western Kenya. In all geographic regions, PrEP use among persons aware that their partner has HIV but are not on ART (SDCs) had a considerable reduction in HIV incidence and was associated with the lowest ICER of scenarios evaluated. PrEP prioritisation for women with multiple partners was the next most efficient strategy, followed by men with multiple partners. Delivering PrEP to AGYW with multiple partners was more efficient than broad PrEP use among AGYW with partners. Strategies of PrEP provision to ABYM were associated with relatively low HIV incidence reductions and high ICERs and therefore unlikely to be efficient. These findings are consistent with previous compartmental modelling studies that find PrEP to be a costly intervention with successful self-selection and targeting needed to increase affordability. One analysis examined PrEP provision across subgroups in 13 countries in SSA and similarly found that PrEP distribution to SDCs was associated with the lowest ICERs, followed by FSWs and then medium-risk AGYW.^15^ A second analysis found that PrEP scale-up to female adolescents (age 15–19 years) in South Africa was associated with the lowest ICERs and targeted strategies for high-risk women and men were more cost-effective than broad provision.^12^ Our analysis uses an individual-based network model which allows for more precise PrEP delivery by partnership characteristics. We use empiric data on the size of subpopulations at risk of HIV and explore geographic heterogeneity in PrEP efficiency.

Our finding that PrEP provision to SDCs is a highly efficient strategy is consistent with the literature and underscores the importance of identifying persons in serodiscordant partnerships, which may be more challenging compared with other subgroups. Leveraging interventions such as assisted partner notification services (APS) and couple HIV testing offer promise in reaching HIV-exposed persons with PrEP. In an APS demonstration project in western Kenya, approximately half of HIV-exposed sexual partners who tested HIV-negative reported HIV risk indicators (eg, multiple partners, recently treated sexually transmitted infection) suggesting they might benefit from PrEP.^31^ Qualitative studies suggest that integrating PrEP into APS can increase index clients’ participation in APS by providing a method to protect their partners and preserve their union.^32^ Similarly, offering PrEP can motivate individuals to uptake couples HIV testing and status disclosure by alleviating concerns of relationship dissolution due to discovery of serodiscordance, as PrEP use can enable the couple to stay together without transmission to the HIV-negative partner.^33^ This strategy is most effective when combined with counselling to encourage the partner with HIV to initiate and adhere to ART. In this analysis, we assumed that knowledge of HIV status did not impact sexual behaviour (eg, condom use). If persons with known HIV increase their condom usage, these ICERs are optimistic.

Similar to previous modelling studies, we find that targeted PrEP provision to persons with multiple partners is a relatively efficient strategy.^13 14^ Empiric data from population-based studies in Africa show that making PrEP broadly available results in large reductions in HIV incidence despite low population coverage, suggesting that individuals are successful at aligning their PrEP use with periods of HIV risk.^5 34^ Interestingly, we find that the counterfactual HIV incidence among men with multiple partners is substantially lower than that of women with multiple partners, but PrEP provision to higher risk men resulted in the highest population-level HIV incidence reduction; this suggests that despite lower HIV prevalence, men may transmit to their partners more frequently than women. Indeed, phylogenetic data from population-based HIV surveillance in Uganda demonstrate an increase in the proportion HIV transmission from men to women over time, with an average age of a transmitting male partner of 34 years.^35^ Therefore, in addition to protecting men’s health, reaching men with HIV prevention is critical to reducing incidence in women. Our finding that PrEP provision to AGYW with multiple partners is less cost-effective compared with older women with multiple partners is unexpected, as the former is a priority group with for PrEP scale-up due to high HIV incidence. This result may be due to heterogeneity within this subgroup; a prior modelling analysis found that PrEP provision to AGYW age 15–19 years averted more HIV incidence than PrEP for AGYW age 20–24 years.^12^ Additionally, PrEP provision to AGYW in our main analysis resulted in a two-fold higher population PrEP prevalence compared with older women (2.26% vs 1.18%). This may indicate that more precise targeting is needed in AGYW to improve PrEP efficiency: for example, PrEP provision to AGYW with older male partners or those who suspect their partner has other partners. Overall, our findings indicate that expanding PrEP prioritisation to include older women is an efficient strategy.

We find that even in geographic settings with high HIV prevalence, broad PrEP provision to all AGYW in partnerships is less efficient than targeted provision; ICERs for broad PrEP use among AGYW were 2.5 times higher than that of targeted AGYW in high HIV prevalence regions and 6–10 times higher in lower prevalence regions. As expected, ICERs for broad PrEP provision were lower in setting of high HIV prevalence but remained less efficient than prioritised provision. Additionally, population-level PrEP coverage in the broad AGYW scenario (8.3%) was much higher than in all others evaluated (eg, 2% in the higher risk AGYW and 1% in women with multiple partner scenarios, respectively) and was associated with a substantially higher budget impact than other strategies. Scaling-up PrEP broadly to AGYW is likely not feasible and cost-prohibitive; therefore, targeted strategies are likely necessary, even in high HIV prevalence settings. Similarly, we find broad PrEP scale-up to ABYM is less efficient than targeted strategies for higher risk ABYM; however, ICERs for both strategies were substantially higher than the corresponding strategies for AGYW. These results are consistent with previous analyses and suggest that PrEP scale-up to young men is less efficient compared with other subgroups.^12^

Our findings highlight the importance of regional differences impacting the efficiency of HIV prevention interventions. For example, comparing two counties with the same baseline HIV prevalence of 3% (Nyamira and Kisii), we found all strategies were more cost-effective in Nyamira. This may be due to underlying differences in population structure, sexual behaviour and age-specific HIV prevalence. Kisii has over twofold greater prevalence of both FSWs and male clients of FSWs, therefore PrEP prioritisation to key populations may be more efficient that provision to the general population (online supplemental table S7). More research is needed to understand the regional differences impacting HIV burden including cultural and socioeconomic factors.

Our results were sensitive to PrEP provision cost and effectiveness (ie, adherence), highlighting the importance of reducing drug costs and supporting adherence to increase efficiency of PrEP scale-up. Additionally, our base case scenarios assumed high PrEP uptake among the prioritised subpopulation; lower uptake resulted in not only lower health benefits but also more cost-effective ICERs. In addition to the payer perspective, we conducted our cost-effectiveness analysis from the societal perspective to reflect the costs incurred by households and society due to HIV care and prevention (eg, transport costs and lost wages associated with accessing PrEP and productivity losses due to HIV). Despite internationally recognised guidelines from the second panel on cost-effectiveness reference case, which recommends utilising the societal perspective, most economic evaluations of HIV interventions assess cost-effectiveness using only the payer perspective.^36^ We find all PrEP scenarios become cost-saving when utilising the societal perspective, which reflects the large economic burden of HIV.

Our findings are subject to several limitations. We do not include the costs of demand generation to reach each group, which likely differ by subpopulation. However, we varied costs in sensitivity analyses to reflect the cost of providing PrEP in community settings (such as pharmacies) and utilising different HIV testing modalities, including HIV self-testing, both of which are promising strategies to increase PrEP scale-up. The cost-of-illness estimates used in this analysis were adjusted from a study in South Africa, which may affect the accuracy of economic calculations. We assume similar adherence across subgroups; if certain groups have higher PrEP adherence, then the relative efficiency of the strategies evaluated would change. Additionally, our model does not include men who have sex with men, persons who inject drugs or other key populations, who have high HIV incidence and can benefit from PrEP. Furthermore, our geographic analysis is limited by the small size of the counties, which resulted in stochastic variation that make some ICER rankings counterintuitive. Additionally, the large number of geographic regions result in many potential calibration parameters, which need to be simultaneously optimised to achieve good model fit. To reduce degrees of freedom, we kept a number of parameter probability constant across regions, which resulted in some geographies more closely fitting empiric HIV prevalence data than others.

Strengths of this analysis include using an individual-based network model, which enabled PrEP targeting to persons with at least one partner. We accounted for parameter uncertainty across 100 good-fitting parameter sets. We employed monthly modelled time steps to simulate more realistic scenarios of short-term PrEP use during periods of HIV risk as observed in PrEP demonstration projects.^37^ We included time-varying HIV test sensitivity to account for the impact of wasted resources (eg, PrEP provision costs) to individuals with HIV who test HIV negative, a concern of widespread PrEP scale-up due to lower test sensitivity in detecting acute HIV infection. We used primary cost data on PrEP provision from microcosting in Kenya.^38^,^40^ Our findings regarding subgroup prioritisation can also be relevant for the implementation of long-acting PrEP products currently in the development pipeline.

## Conclusions

Overall, we find that PrEP scale-up can avert a substantial number HIV infections and implementing demand-generation strategies for subgroups at higher risk can increase the efficiency of PrEP programmes. Our results on the health and cost impact of PrEP scale-up across geographic regions in western Kenya can be used for budgetary planning and priority setting.

## supplementary material

10.1136/bmjgh-2024-015835 online supplemental file 1

## Data Availability

Data are available in a public, open access repository. Data are available upon reasonable request. All data relevant to the study are included in the article or uploaded as supplementary information.
