# Supplementary material for: Health and economic impact of oral PrEP provision across subgroups in western Kenya: a modelling analysis
Source: BMJ Glob Health. 2025 Jan 11;10(1):e015835. doi: 10.1136/bmjgh-2024-015835 (PMC11749867; doi:10.1136/bmjgh-2024-015835)
Supplement: online supplemental file 1 [file bmjgh-10-1-s001.pdf]

Supplemental Appendix accompanying the manuscript: "Health and economic impact of oral PrEP provision across subgroups in western Kenya: a modeling analysis"

Rachel Wittenauer, Linxuan Wu, Sarah N Cox, Brian Pfau, Monisha Sharma

Contents

Section 1 | Model Calibration and Key Parameters **1**
1.0 Model structure overview 1
1.1 Model calibration to HIV data 1
Table S1a. HIV prevalence in counties of the former Nyanza province, by age and gender¥ 1
Table S1b. HIV prevalence among men and women ages 15-49, by county and gender¥ 2
Table S2. Number of people on ART by county, gender, and age group¥ 3
Figure S1. Model fit to age-specific and overall prevalence from population-based surveys by sex 4
1.2 Model calibration to demographic data 9
Table S3. Population size by gender, county, and age group in 2019¥ 10
Table S4a. Age-specific population fertility rates in Kenya 1950-2049¥ 11
Table S4b. Age-specific HIV-deleted mortality rates among in Kenya 1950-2049 by gender ¥ 12
Figure S2. Model predicted population growth by sex 13
1.3 Key model inputs and parameters 14
Table S5. Key model parameters. 14
Table S6. Circumcision status quo by county, age group, and year ¥ 17
Table S7: Lifetime probability of becoming a female sex worker or male client of FSW in western Kenya by county 18

Section 2 | Interventions and Analysis Design **19**
2.1 Cost approach and calculations 19
Table S8. Cost parameter calculations¥ 20
2.2 Intervention design 22
Table S9. Implementation scenario definitions 22
Table S10. Assumed RDT sensitivity 22
Figure S3. Schematic depicting model implementation of PrEP delivery 23
Table S11. Summary of key PrEP implementation assumptions 23

Section 3 | Results and Sensitivity Analyses **24**
3.1 Additional main analysis results 24
Table S12. PrEP outcomes by scenario in western Kenya (Median, 90% CI)* 24
Figure S4. PrEP initiations by scenario 25
Figure S5. HIV Incidence Among Ages 18-49 (2020 - 2027) 26
Table S13. Proportion of scenarios that are cost-effective by county 26
Figure S6. Cost-effectiveness plane of main analysis results 27
Figure S7a. Components of 20-year total cost relative to baseline by category in each scenario 28
Figure S7b. Total 20-year discounted costs by category in each scenario 28
Figure S7c. Proportions of total 20-year discounted costs by category in each scenario 29
Table S14a. Total 20-year discounted costs by category in each scenario 29
Table S14b. Incremental 20-year discounted costs by category in each scenario versus baseline 30
Figure S8a. Total 5-year undiscounted costs by category in each scenario 30
Figure S8b. Proportions of 5-year undiscounted costs by category in each scenario 31
3.2 Sensitivity Analyses 32
Table S15. Summary of sensitivity analyses 32
Table S16. ICER results of one-way sensitivity analyses 32

Table S17. PrEP outcomes by scenario (Median, 90% CI) for Lower PrEP Coverage* 33
Table S18. ICERs by scenario and geography for Lower PrEP Coverage 33
Table S19. Societal perspective costs and calculations 34
Figure S9. Cost-effectiveness plane of societal perspective results 35

Supplemental References 36

Section 1 | Model Calibration and Key Parameters

1.0 Model structure overview

EMOD is an open-source agent-based model developed by the Institute for Disease Modeling (IDM) within the Bill & Melinda Gates Foundation. Documentation is available extensively on the IDM website (https://docs.idmod.org/projects/emod-hiv/en/latest/) [1].

Model structure
EMOD is an agent-based stochastic simulation model of heterosexual and vertical HIV transmission.[2] For this analysis, we calibrated the model to population and HIV epidemic of western Kenya comprised of six counties, each with different population structures and HIV prevalences and which are structured as compartments in the model assuming no migration between counties. Individuals can form partnerships, have coital acts, and access the HIV prevention and care cascade including voluntary medical male circumcision, HIV testing and HIV treatment. Partnership formation is parameterized by sexual behavior data from demographic health surveys including age differences, number of partners and engagement in transactional sex. The model is subsequently calibrated to match age specific HIV prevalence. Partnerships are classified into four types: transitory, informal, marital, and commercial. More detail is available in prior publications.[1–5]

1.1 Model calibration to HIV data

Model calibration
The model was calibrated to empiric data including HIV prevalence by sex, age, and year, and number on ART by sex, age, and year. The quality of fit was summarized with a likelihood score, and calibration was then performed using Incremental Mixture Importance Sampling as described in previous papers using this model and on the IDM model documentation website.[2, 4, 6]For model calibration to HIV-related data, the HIV prevalence data is summarized in **Table S1a** and **Table S1b**, the number on ART is summarized in **Table S2**, and the model fit to these data are summarized in **Figure S1** parts a, b, and c (all below).

The model displays close fits to overall HIV prevalence across age groups (See **Figure S1**). Additionally, the majority of the modeled age-specific HIV prevalence falls within the 95% confidence interval of empirical estimates. The model tends to underestimate HIV prevalence among the two oldest age groups for both males and females (age 40-44, 45-49). Among females, the model's estimates align well with the empiric data for the youngest age groups (age 15-19, 20-24), but tends to overestimate HIV prevalence among those aged 25-29 and 30-34. For males, the model tends to overestimate HIV prevalence for the two youngest age groups and underestimate the 25-29 age group. There is considerable uncertainty in empiric age specific estimates of HIV prevalence.

Table S1a. HIV prevalence in counties of the former Nyanza province, by age and gender[¥]

Age group	2003		2007		2008		2012		2018	
	Men	Women	Men	Women	Men	Women	Men	Women	Men	Women
15 - 19	0.0015	0.0459	0.0121	0.0773	0.0184	0.1078	0.0151	0.0486	0.0025	0.0294
20 - 24	0.0562	0.2997	0.0257	0.2056	0.0578	0.1201	0.0289	0.1411	0.0216	0.0910
25 - 29	0.2429	0.2301	0.1956	0.2454	0.2450	0.2228	0.2107	0.2454	0.0705	0.2334
30 - 34	0.1840	0.1632	0.2578	0.2576	0.1530	0.2593	0.2392	0.2047	0.0911	0.2696
35- 39	0.2064	0.1838	0.2384	0.2227	0.2275	0.2259	0.1955	0.2811	0.1675	0.2705
40 - 44	0.2533	0.3500	0.2024	0.1799	0.2501	0.0927	0.3132	0.1694	0.2075	0.2765
45 - 49	0.1624	0.1651	0.2103	0.1291	0.1331	0.1716	0.1623	0.2287	0.2796	0.1991
15 - 49	0.1160	0.1830	0.1140	0.1760	0.1140	0.1600	0.1340	0.1760	0.0826	0.1667

[¥]**Sources:** Kenya Demographic and Health Surveys, 2003 & 2008; Kenya AIDS Indicator Surveys, 2007 & 2012; Kenya Population-based HIV Impact Assessment 2018. The counties of the former Nyanza province are Homa Bay, Kisii, Kisumu, Migori, Nyamira, and Siaya.

Table S1b. HIV prevalence among men and women ages 15-49, by county and gender[¥]

County	2003		2007		2008		2012		2018	
	Men	Women	Men	Women	Men	Women	Men	Women	Men	Women
Homa Bay	0.1097	0.2458	0.2514	0.3259	0.1737	0.2524	0.2217	0.2787	0.1279	0.2532
Kisii	0.0114	0.0853	0.0445	0.0693	0.0330	0.0573	0.0346	0.0368	0.0458	0.0684
Kisumu	0.1663	0.1914	0.1139	0.1847	0.1109	0.1810	0.1940	0.2022	0.0960	0.2096
Migori	0.1804	0.1860	0.1685	0.2181	0.1923	0.2228	0.1435	0.1925	0.0706	0.1758
Nyamira	0.0029	0.0742	-	-	0.0234	0.0544	0.0419	0.1045	0.0247	0.0432
Siaya	0.1824	0.2424	0.1445	0.2130	0.1526	0.1921	0.2596	0.2990	0.0961	0.1905

[¥]**Sources:** Kenya Demographic and Health Surveys, 2003 & 2008; Kenya AIDS Indicator Surveys, 2007 & 2012; Kenya Population-based HIV Impact Assessment 2018.

Table S2. Number of people on ART by county, gender, and age group[¥]

Gender	County	Age group	Year													
			2004	2005	2006	2007	2008	2009	2010	2011	2012	2013	2014	2015	2016	2017
Men	Homa Bay	0 - 14	-	-	-	-	-	-	-	-	-	-	2,945	3,583	4,109	4,192
		15 - 99	1,067	2,313	5,148	7,194	10,002	14,436	17,178	15,954	17,522	18,279	19,157	22,834	26,441	29,220
	Kisii	0 - 14	-	-	-	-	-	-	-	-	-	-	828	993	1,109	1,083
		15 - 99	-	-	-	-	-	-	-	2,972	-	-	4,614	5,451	6,604	7,169
	Kisumu	0 - 14	-	-	-	-	-	-	-	-	-	-	3,101	3,245	3,525	3,607
		15 - 99	945	2,047	4,557	6,368	8,853	12,779	15,206	14,122	15,511	16,180	21,216	24,550	28,082	31,021
	Migori	0 - 14											2,309	2,295	2,678	2,673
		15 - 99	711	1,541	3,430	4,793	6,664	9,619	11,446	10,630	11,675	12,179	13,929	15,165	17,438	18,455
	Nyamira	0 - 14	-	-	-	-	-	-	-	-	-	-	484	552	578	611
		15 - 99	-	-	-	-	-	-	-	1,362	-	-	2,120	2,585	3,142	3,474
	Siaya	0 - 14	-	-	-	-	-	-	-	-	-	-	2,645	2,950	3,017	3,197
		15 - 99	860	1,864	4,148	5,797	8,060	11,633	13,843	12,856	14,120	14,730	16,163	18,611	21,477	23,762
Women	Homa Bay	0 - 14	-	-	-	-	-	-	-	-	-	-	3,431	3,835	4,426	4,535
		15 - 99	1,359	2,944	6,551	9,155	12,522	17,202	21,798	31,454	35,182	38,819	40,118	49,956	57,286	61,811
	Kisii	0 - 14	-	-	-	-	-	-	-	-	-	-	906	1,079	1,200	1,146
		15 - 99	-	-	-	-	-	-	-	7,902	-	-	11,691	14,350	17,274	19,044
	Kisumu	0 - 14	-	-	-	-	-	-	-	-	-	-	3,241	3,393	3,810	3,831
		15 - 99	1,203	2,606	5,799	8,104	11,084	15,227	19,296	27,843	31,143	34,362	41,230	48,424	56,384	60,789
	Migori	0 - 14	-	-	-	-	-	-	-	-	-	-	2,526	2,448	2,868	2,884
		15 - 99	905	1,961	4,365	6,100	8,343	11,461	14,524	20,958	23,442	25,865	27,896	31,964	38,637	40,891
	Nyamira	0 - 14	-	-	-	-	-	-	-	-	-	-	506	567	601	622
		15 - 99	-	-	-	-	-	-	-	3,766	-	-	5,964	7,210	8,258	8,654
	Siaya	0 - 14	-	-	-	-	-	-	-	-	-	-	2,778	3,136	3,299	3,569
		15 - 99	1,095	2,372	5,279	7,377	10,090	13,862	17,566	25,347	28,351	31,281	33,911	39,853	44,892	48,808
Both	All	15-99	8,954	19,404	43,185	60,350	83,142	116,787	143,877	175,003	194,552	210,769	246,293	301,029	333,333	389,159

[¥]Source: Kenya Ministry of Health

Figure S1. Model fit to age-specific and overall prevalence from population-based surveys by sex

* Blue curves refer to the LOESS line fitting to the 100 simulations; the error bars refer to the empirical estimates and 95% confidence intervals for HIV prevalence obtained from Kenya Demographic and Health Surveys and Kenya AIDS Indicator Surveys.

Figure S1a. HIV Prevalence by Age across all 6 Counties

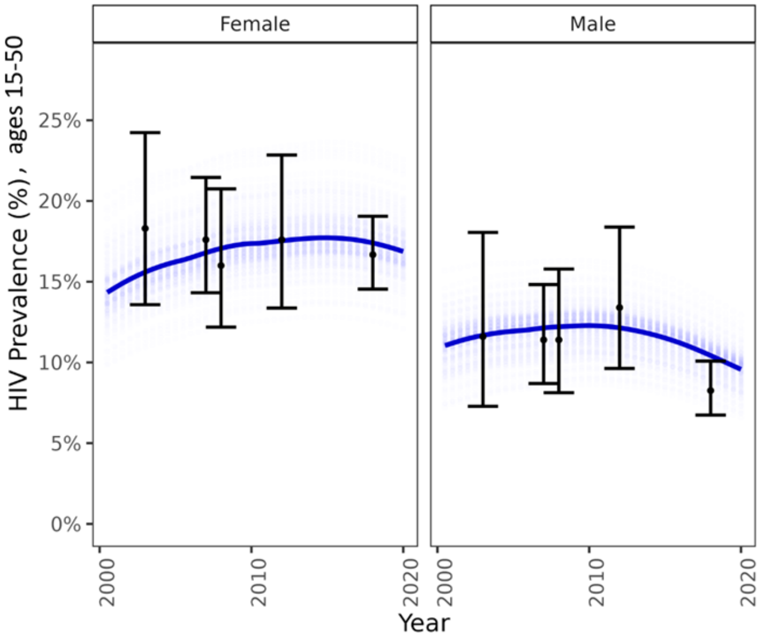

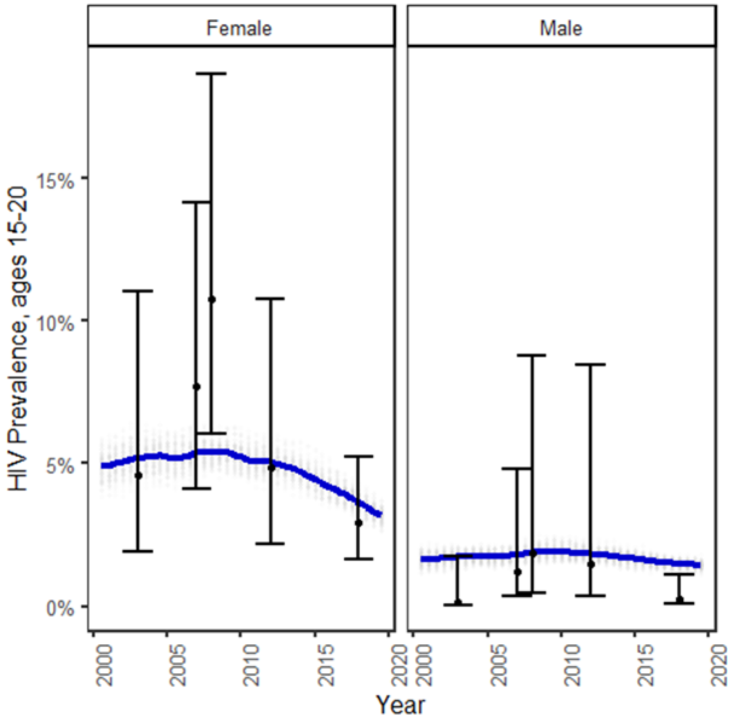

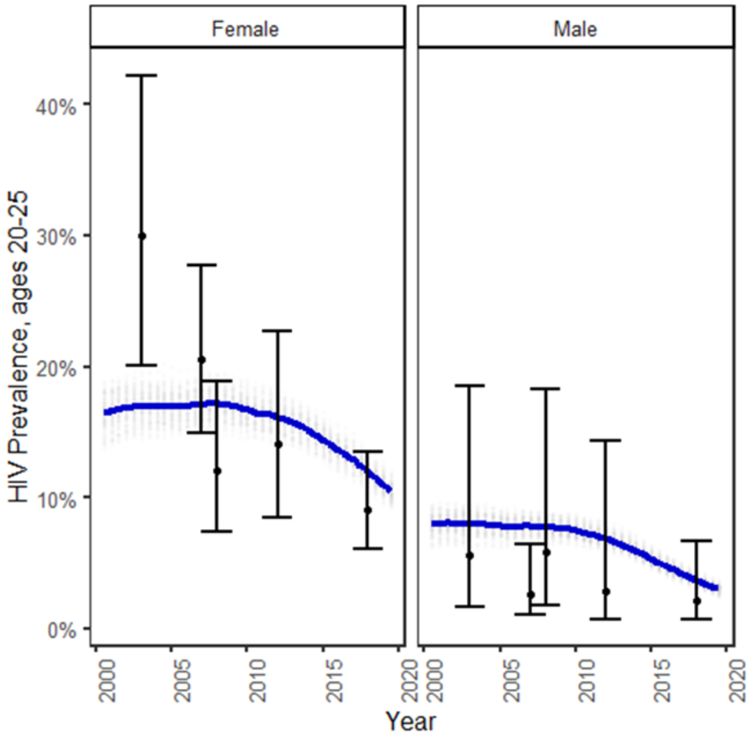
Female
Male
HIV Prevalence, ages 20-25
40%
30%
20%
10%
0%
2000
2005
2010
2015
2020
Year

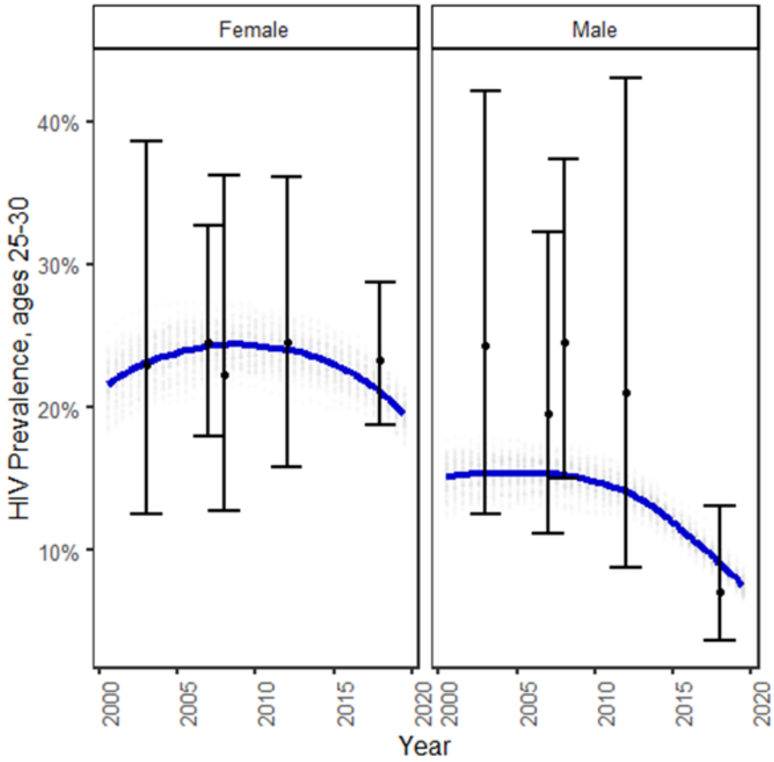
Female
Male
HIV Prevalence, ages 25-30
40%
30%
20%
10%
2000
2005
2010
2015
2020
Year

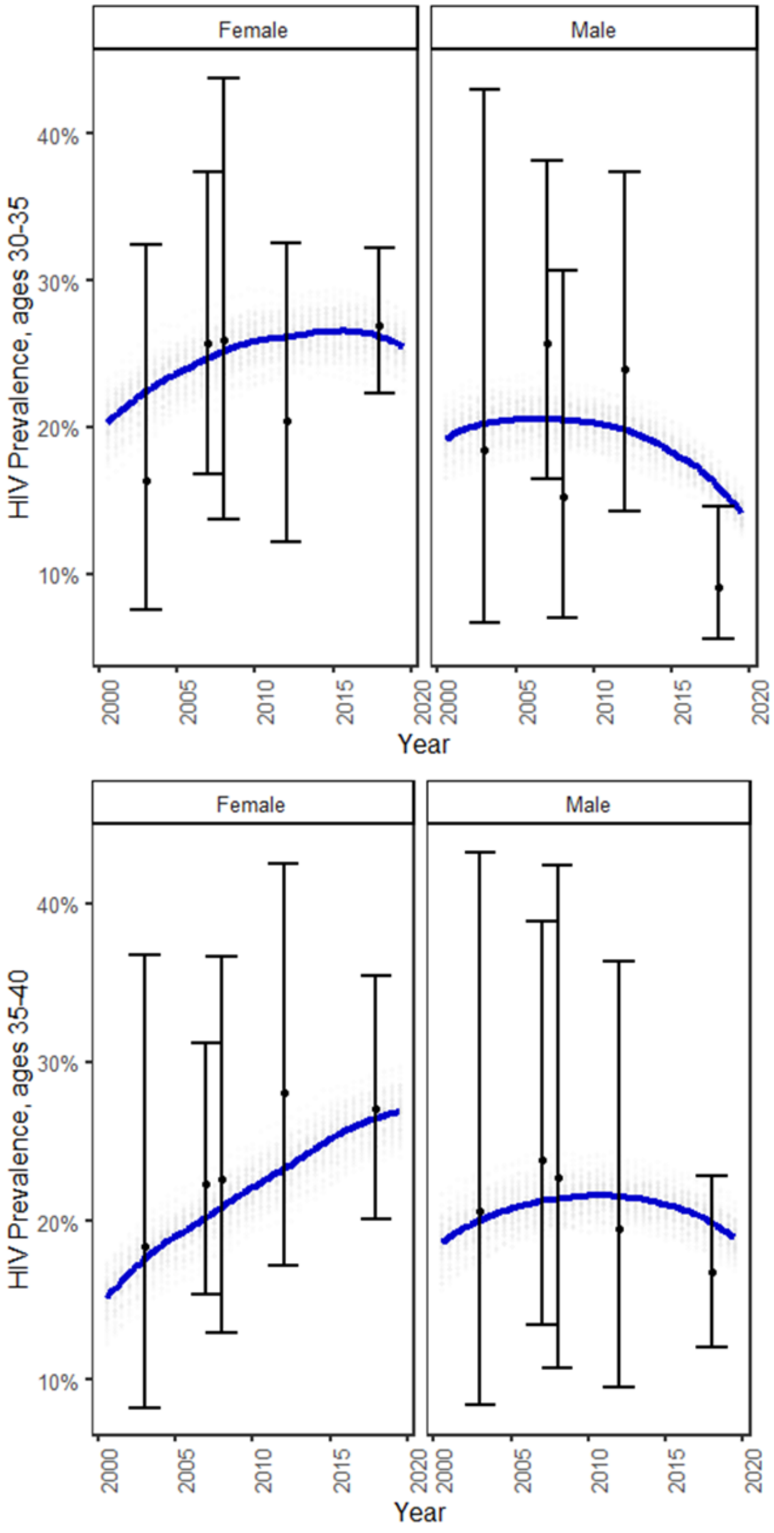
Female
Male
HIV Prevalence, ages 30-35
40%
30%
20%
10%
2000
2005
2010
2015
2020
Year
Female
Male
HIV Prevalence, ages 35-40
40%
30%
20%
10%
2000
2005
2010
2015
2020
Year

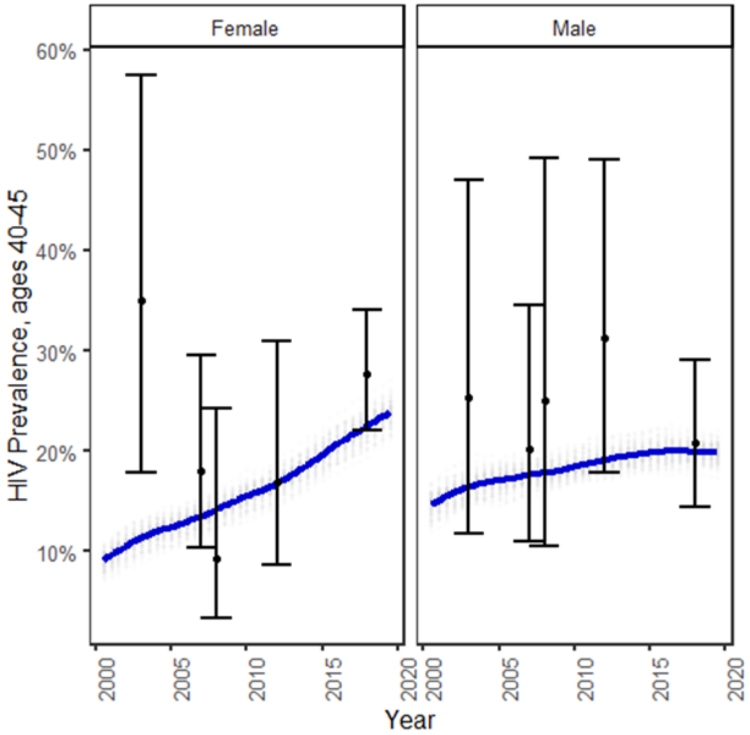
Female
Male
HIV Prevalence, ages 40-45
60%
50%
40%
30%
20%
10%
2000
2005
2010
2015
2020
Year

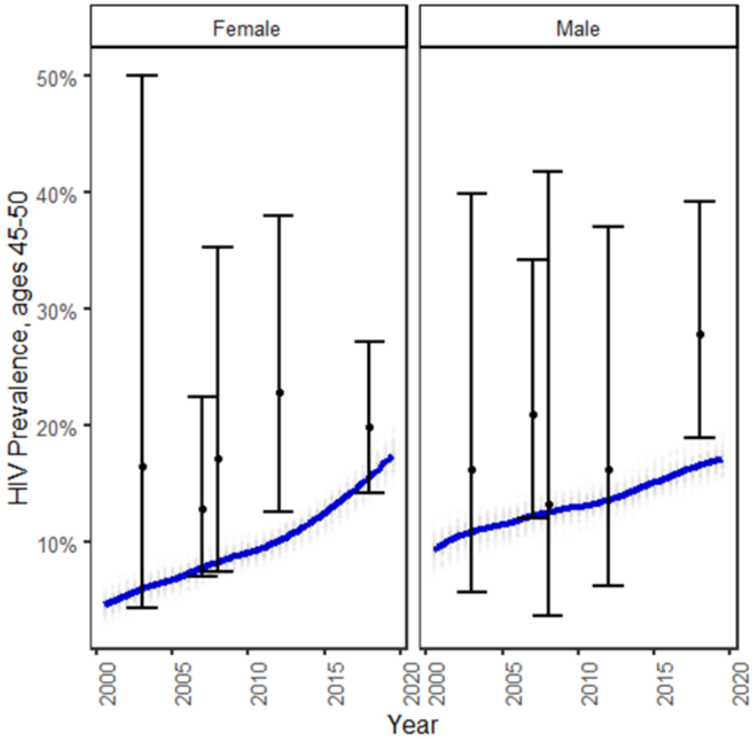
Female
Male
HIV Prevalence, ages 45-50
50%
40%
30%
20%
10%
2000
2005
2010
2015
2020
Year

Figure S1b. HIV Prevalence in people ages 15-50 years, by County

1. Homa Bay

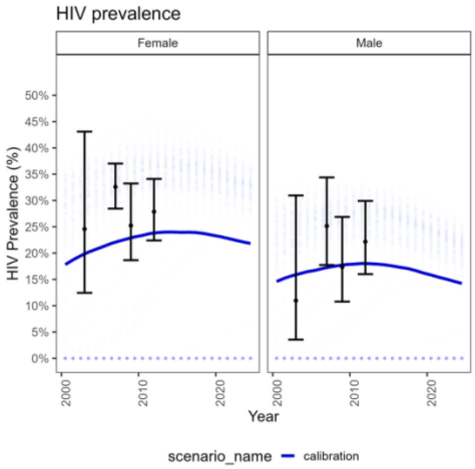

2. Kisii

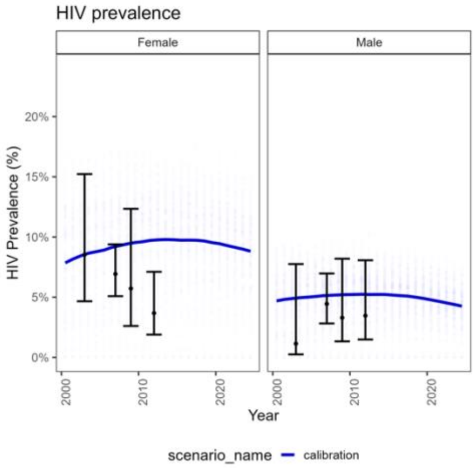

3. Kisumu

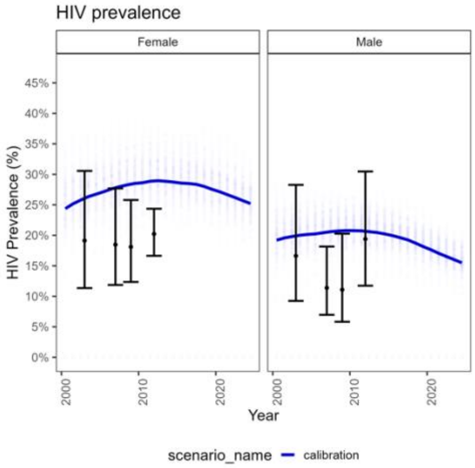

4. Migori

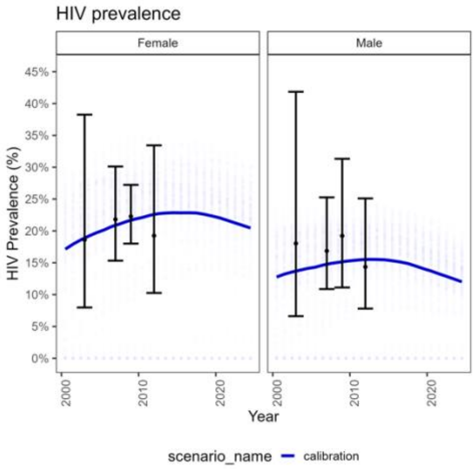

5. Nyamira

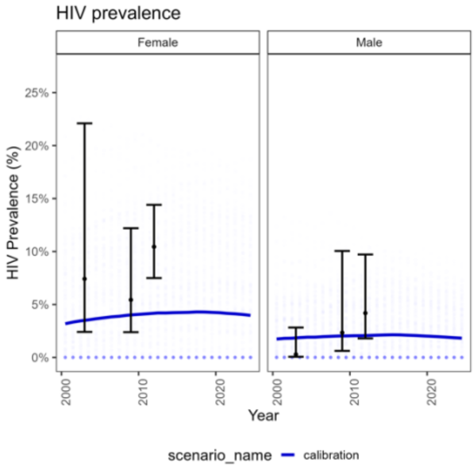

6. Siaya

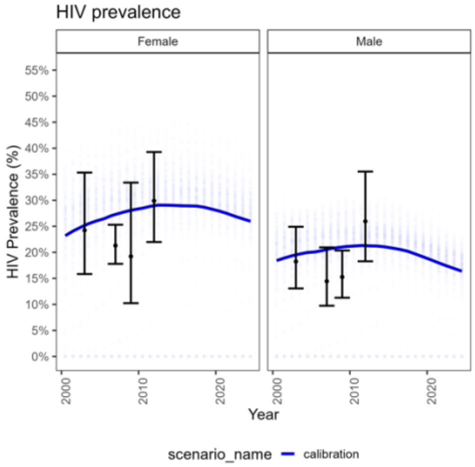

Figure S1c. Number on ART

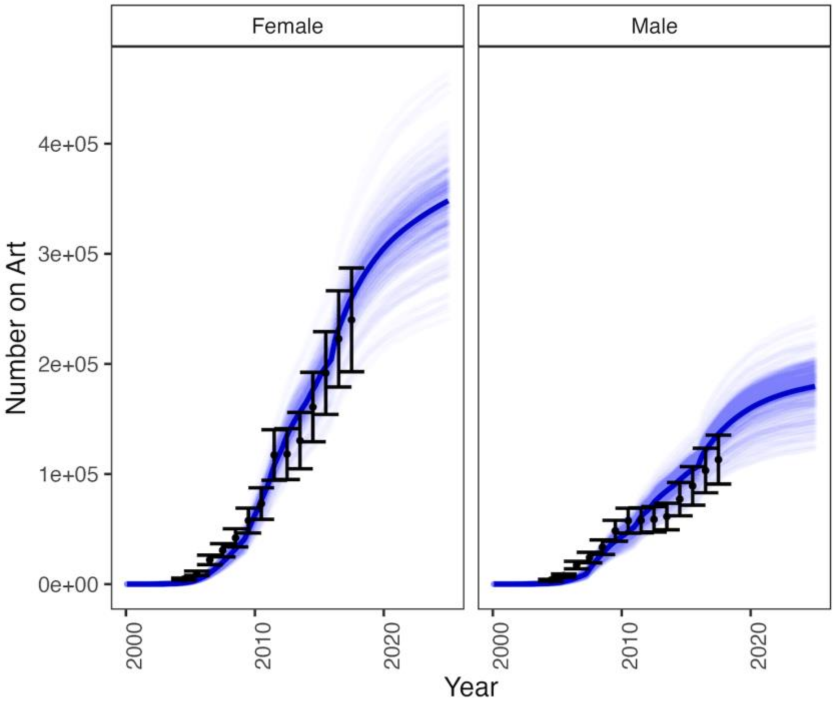

1.2 Model calibration to demographic data

<u>Population scaling factor</u>
We applied scaling factors of approximately 60 to each run to increase the size of our modeled population and outcomes to reflect the actual population size in western Kenya. To determine the scaling factor for each simulation, we divided the size of the Kenya 2019 census population by the size of the population in 2019 and multiplied this ratio by the modeled population and outcomes. The size of the scaling factor varied slightly depending on model run due to stochastic variability in modeled population size.
For model calibration and validation to population, fertility and mortality data, see **Table S3, Table S4a,** and **Table S4b**. For the overall fit of the model's total population to these demographic data see **Figure S2**.

Table S3. Population size by gender, county, and age group in 2019[¥]

Age Group	Men						Women					
	Homa Bay	Kisii	Kisumu	Migori	Nyamira	Siaya	Homa Bay	Kisii	Kisumu	Migori	Nyamira	Siaya
0 - < 1	18,335	18,236	17,457	19,265	10,313	15,093	18,354	17,993	16,926	19,309	10,263	14,860
1 - 4	69,799	69,529	63,054	69,921	41,165	56,269	69,250	69,023	63,172	69,519	40,396	55,901
5 - 9	75,926	76,757	67,083	73,872	46,450	60,966	75,973	75,778	67,779	74,333	46,867	60,710
10 - 14	68,689	68,473	62,706	64,300	42,590	58,296	67,159	68,072	63,359	63,249	42,198	56,248
15 - 19	57,430	59,228	55,597	53,075	36,604	49,220	54,119	60,776	56,741	52,238	36,786	47,825
20 - 24	39,573	41,898	47,281	38,690	24,409	32,725	50,309	58,225	57,649	48,004	34,184	41,443
25 - 29	30,437	32,792	40,964	30,727	19,515	25,961	36,016	42,878	40,614	34,670	27,273	30,135
30 - 34	23,259	26,678	30,412	23,344	16,605	20,359	26,342	30,031	27,515	25,630	19,487	22,328
34 - 39	16,013	21,766	21,251	17,024	14,039	14,793	20,010	26,051	20,611	19,313	17,106	17,932
40 - 44	11,914	15,718	15,145	12,170	10,470	11,118	16,513	18,360	16,894	14,773	11,377	16,082
45 - 49	11,124	16,797	13,361	10,549	11,318	10,390	15,248	19,181	15,298	12,888	11,886	15,486
50 - 54	9,705	12,789	11,251	8,565	8,379	9,079	12,942	14,136	12,504	10,314	8,703	14,541
55 - 59	8,159	9,527	8,718	6,399	5,999	8,414	9,833	9,528	9,175	7,692	5,819	12,265
60 - 64	6,989	7,395	7,054	5,250	5,026	7,712	8,587	7,654	7,597	6,000	5,107	11,081
65 - 69	4,325	4,637	4,163	3,382	3,094	5,107	5,957	5,320	5,402	4,508	3,322	7,732
70 - 74	4,029	3,945	3,777	2,907	2,753	5,175	5,355	5,017	4,757	3,524	3,153	7,173
75 - 79	2,835	2,743	2,392	2,033	1,778	3,549	3,891	3,338	3,356	2,968	1,919	5,464
80 - 99	3,726	3,701	2,821	2,624	2,393	4,159	5,316	5,891	4,615	3,636	3,475	6,155

[¥]Source: Kenya National Bureau of Statistics, 2019 Census

Table S4a. Age-specific population fertility rates in Kenya 1950-2049[¥]

	Age-specific fertility rates (births per 1,000 women)						
Year	15-19	20-24	25-29	30-34	35-39	40-44	45-49
1950-1955	169.1	351.6	338.1	284.3	203.5	110.7	38.9
1955-1960	175.9	365.9	351.9	295.8	211.8	115.2	40.5
1960-1965	182.3	379.1	364.5	306.5	219.4	119.4	41.9
1965-1970	183.3	381.2	366.6	308.2	220.6	120	42.2
1970-1975	180.6	375.5	361.1	303.6	217.3	118.3	41.5
1975-1980	172.7	359.1	345.3	290.3	207.8	113.1	39.7
1980-1985	163.1	339.2	326.2	274.2	196.3	106.8	37.5
1985-1990	147.8	307.3	295.5	248.4	177.8	96.8	34.0
1990-1995	115.3	268.9	252.0	206.8	161.6	73.4	52.0
1995-2000	111.5	260.7	253.3	196.2	143.3	62.4	42.7
2000-2005	104.2	243.6	236.7	183.4	133.9	58.3	39.9
2005-2010	97.1	227.1	221.4	170.6	123.7	53.6	36.5
2010-2015	86.2	201.9	202.3	149.2	102.1	42.4	27.9
2015-2020	75.1	176.5	179.8	129.6	85.8	34.8	22.4
2020-2024	69.9	165.1	171.8	120.2	76.2	29.9	18.6
2025-2029	65.0	154.7	164.8	112.6	68.5	26.0	15.5
2030-2034	60.6	145.7	159.1	106.7	62.5	22.9	13.1
2035-2039	56.2	137.2	153.6	101.8	57.6	20.4	11.0
2040-2044	52.2	129.7	149.3	98.2	53.8	18.5	9.4

[¥]**Source**: 2019 World Population Prospects

Table S4b. Age-specific HIV-deleted mortality rates among in Kenya 1950-2049 by gender ¥

In EMOD, we modelled HIV cause-deleted mortality rates in the background and HIV transmission and related morality rates in the foreground. To calculate the HIV deleted mortality, we first investigated all-cause mortality trends between 1960 and 2000 between countries with and without widespread HIV-AIDS epidemic. Countries without the epidemic demonstrated an exponential decline in mortality, while those grappling with the epidemic experienced an exponential decrease interrupted by a sudden spike in the 1980s. We assumed the difference between these two curves (i.e., the spike) is due to the impact of the HIV-AIDS epidemic. In Kenya, we fitted an exponential curve from 1970 to 1980 to represent the cause-deleted mortality. We then conducted a check on the population demographics generated by EMOD, ensuring that the age structure of the population simulated through both cause-deleted mortality and simulated HIV transmission aligns with the UN WPP's population projections post-1980. The units presented are percentage mortality per person-year.

		Age-specific HIV-deleted mortality rates (%) (age groups in years)						
Sex	Year	15-19	20-24	25-29	30-34	35-39	40-44	45-49
Women	1997.5	0.136	0.191	0.244	0.294	0.361	0.453	0.542
	2002.5	0.116	0.167	0.215	0.259	0.321	0.408	0.493
	2007.5	0.100	0.145	0.188	0.229	0.286	0.367	0.449
	2012.5	0.085	0.127	0.166	0.202	0.254	0.331	0.408
	2017.5	0.073	0.110	0.145	0.178	0.226	0.298	0.371
	2022.5	0.063	0.096	0.128	0.158	0.201	0.268	0.338
	2027.5	0.054	0.084	0.112	0.139	0.179	0.241	0.307
	2032.5	0.046	0.073	0.099	0.123	0.160	0.217	0.280
	2037.5	0.039	0.064	0.087	0.108	0.142	0.196	0.254
	2042.5	0.034	0.055	0.076	0.096	0.126	0.176	0.231
Men	1997.5	0.165	0.253	0.288	0.345	0.433	0.551	0.713
	2002.5	0.142	0.219	0.251	0.304	0.385	0.494	0.648
	2007.5	0.121	0.189	0.219	0.267	0.343	0.443	0.589
	2012.5	0.104	0.163	0.191	0.235	0.305	0.398	0.535
	2017.5	0.089	0.141	0.166	0.207	0.271	0.357	0.486
	2022.5	0.076	0.122	0.145	0.182	0.241	0.320	0.441
	2027.5	0.065	0.106	0.126	0.160	0.215	0.287	0.401
	2032.5	0.056	0.091	0.110	0.141	0.191	0.258	0.364
	2037.5	0.048	0.079	0.096	0.124	0.170	0.231	0.331
	2042.5	0.041	0.068	0.083	0.109	0.151	0.208	0.300

¥**Source**: 2019 World Population Prospects

Figure S2. Model predicted population growth by sex

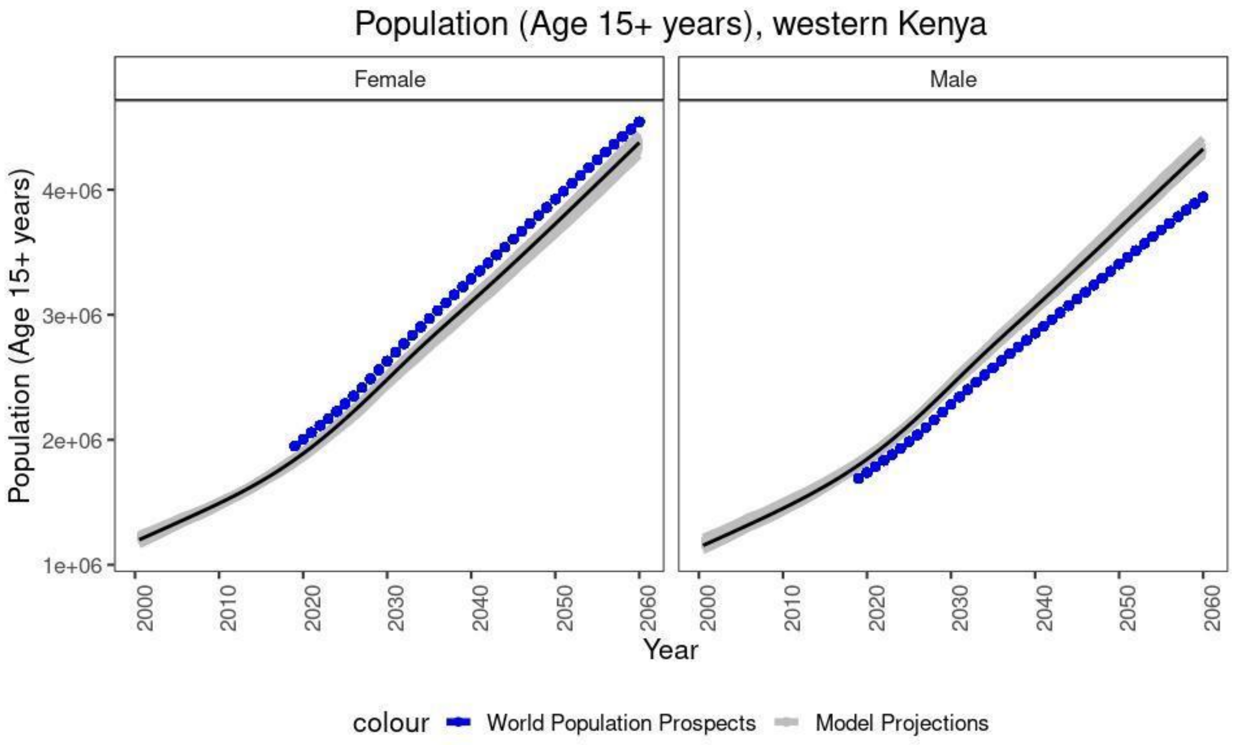

Source: 2019 population for former Nyanza province based on the Kenya Population and Housing Census. Annual growth rates based on World Population Prospects, assuming growth in the former Nyanza province is similar to national growth rate.

1.3 Key model inputs and parameters

Table S5. Key model parameters.

Select model parameters used to fit the EMOD-HIV transmission model to survey data on prevalence and ART coverage from Kenya. Median and interquartile ranges (IQRs) reported for all dynamic parameters used in the calibration process from 100 best-fitting parameter sets. †

Parameter	Parameter Description	Fitted median	(IQR)
ARTLinkMax	Maximum probability of linkage to ART	0.999	(0.978, 1.000)
ARTLinkMid	Year of ART linkage (given eligibility), that is, time of the inflection point in the sigmoid trend.	2003.075	(2002.9945, 2003.2309)
CircumcisionReducedAcquire	The reduction of susceptibility to HIV by voluntary male medical circumcision (VMMC).	0.600	(0.598, 0.600)
Homa_BayInfrmlCondomsMax	Maximum rate of condom use in informal relationships in Homa Bay	0.226	(0.221, 0.231)
Homa_BayLOWRisk	Proportion of the population that is low-risk in Homa Bay	0.574	(0.567, 0.588)
Homa_BayTrnsCondomsMax	Maximum rate of condom use in transitory relationships in Homa Bay	0.233	(0.223, 0.242)
InfmrlFormRate	Informal relationship formation rate	0.00009	(0.00009, 0.00011)
InfrmlCondomMid	Year midpoint of logistic scale-up of condom use in informal relationships	1998.426	(1998.228, 1998.708)
InfrmlCondomRate	Rate of logistic scale-up of condom use in informal relationships	1.774	(1.745, 1.816)
InfrmlCondomsMax	Maximum rate of condom use in informal relationships	0.225	(0.217, 0.231)
InfrmlDurHet	Heterogeneity in duration of informal relationships	0.750	(0.750, 0.750)
KisiiInfrmlCondomsMax	Maximum rate of condom use in informal relationships in Kisii	0.219	(0.212, 0.226)
KisiiLOWRisk	Proportion of the population that is low-risk in Kisii	0.947	(0.943, 0.950)
KisiiTrnsCondomsMax	Maximum rate of condom use in transitory relationships in Kisii	0.357	(0.346, 0.366)
KisumuInfrmlCondomsMax	Maximum rate of condom use in informal relationships in Kisumu	0.179	(0.167, 0.185)
KisumuLOWRisk	Proportion of the population that is low-risk in Kisumu	0.764	(0.762, 0.765)
KisumuTrnsCondomsMax	Maximum rate of condom use in transitory relationships in Kisumu	0.347	(0.341, 0.353)
MaleToFemaleOld	Male-to-female relative risk of infection among older individuals	1.567	(1.535, 1.674)
MaleToFemaleYoung	Male-to-female relative risk of infection among young individuals	1.229	(1.175, 1.333)
MaxInfrmlFLOW	Maximum number of informal relationships among low-risk females	1.230	(1.208, 1.242)
MaxInfrmlFMED	Maximum number of informal relationships among medium-risk females	2.498	(2.466, 2.543)
MaxInfrmlMLOW	Maximum number of informal relationships among low-risk males	1.110	(1.086, 1.117)
MaxInfrmlMMED	Maximum number of informal relationships among medium-risk males	2.530	(2.442, 2.735)
MaxMrtlFMED	Maximum number of marital relationship among medium-risk females	1.118	(1.104, 1.136)
MaxMrtlMMED	Maximum number of marital relationship among medium-risk males	1.335	(1.309, 1.356)

Parameter	Parameter Description	Fitted median	(IQR)
MaxTrnsFLOW	Maximum number of transitory relationships among low-risk females	1.646	(1.619, 1.663)
MaxTrnsFMED	Maximum number of transitory relationships among medium-risk females	2.993	(2.956, 3.000)
MaxTrnsMLOW	Maximum number of transitory relationships among low-risk males	1.711	(1.701, 1.730)
MaxTrnsMMED	Maximum number of transitory relationships among medium-risk males	3.000	(2.956, 3.000)
MigoriInfrmlCondomsMax	Maximum rate of condom use in informal relationships in Migori	0.205	(0.199, 0.212)
MigoriLOWRisk	Proportion of the population that is low-risk in Migori	0.784	(0.779, 0.794)
MigoriTrnsCondomsMax	Maximum rate of condom use in transitory relationships in Migori	0.257	(0.250, 0.268)
MrtlCondomMax	Maximum rate of condom use in marital relationships	0.189	(0.184, 0.192)
MrtlCondomMid	Year midpoint of logistic scale-up of condom use in marital relationships	1995.815	(1995.656, 1996.058)
MrtlCondomRate	Rate of logistic scale-up of condom use in marital relationships	2.125	(2.062, 2.188)
MrtlFormRate	Marital relationship formation rate	0.000056	(0.000055, 0.000061)
NyamiraInfrmlCondomsMax	Maximum rate of condom use in informal relationships in Nyamira	0.109	(0.106, 0.122)
NyamiraLOWRisk	Proportion of the population that is low-risk in Nyamira	0.928	(0.922, 0.935)
NyamiraTrnsCondomsMax	Maximum rate of condom use in transitory relationships in Nyamira	0.297	(0.288, 0.304)
PrExInfrmlFemLOW	Probability of potential for extra-relational informal relationship among low-risk females	0.366	(0.364, 0.371)
PrExInfrmlFemMED	Probability of potential for extra-relational informal relationship among medium-risk females	0.357	(0.352, 0.364)
PrExInfrmlMaleLOW	Probability of potential for extra-relational informal relationship among low-risk males	0.244	(0.222, 0.249)
PrExInfrmlMaleMED	Probability of potential for extra-relational informal relationship among medium-risk males	0.394	(0.388, 0.405)
PrExTrnsFemLOW	Probability of potential for extra-relational transitory relationship among low-risk females	0.247	(0.240, 0.255)
PrExTrnsFemMED	Probability of potential for extra-relational transitory relationship among medium-risk females	0.460	(0.455, 0.486)
PrExTrnsMaleLOW	Probability of potential for extra-relational transitory relationship among low-risk males	0.318	(0.311, 0.327)
PrExTrnsMaleMED	Probability of potential for extra-relational transitory relationship among medium-risk males	0.633	(0.623, 0.646)
PreARTLinkMax	Maximum probability of linkage to pre-ART care	0.737	(0.724, 0.754)
PreARTLinkMid	Year midpoint of logistic scale-up of pre-ART linkage	2003.077	(2002.659, 2003.212)
PreARTLinkMin	Minimum probability of linkage to pre-ART care	0.404	(0.393, 0.424)
RiskAssortivity	Risk assortivity	0.663	(0.652, 0.674)
SeedYrHigh	Seed year	1982	(1982.000, 1982.000)
SexualDebutAgeFemaleWeibullHeterogeneity	Heterogeneity parameter of Weibull distribution of female age of sexual debut	0.085	(0.079, 0.091)
SexualDebutAgeFemaleWeibullScale	Scale parameter of Weibull distribution of female age of sexual debut	16.085	(16.006, 16.185)
SexualDebutAgeMaleWeibullHeterogeneity	Heterogeneity parameter of Weibull distribution of male age of sexual debut	0.043	(0.040, 0.049)

Parameter	Parameter Description	Fitted median	(IQR)
SexualDebutAgeMaleWeibullScale	Scale parameter of Weibull distribution of male age of sexual debut	16.003	(15.940, 16.065)
SiayaInfrmlCondomsMax	Maximum rate of condom use in informal relationships in Siaya	0.146	(0.140, 0.152)
SiayaLOWRisk	Proportion of the population that is low-risk in Siaya	0.723	(0.717, 0.734)
SiayaTrnsCondomsMax	Maximum rate of condom use in transitory relationships in Siaya	0.321	(0.307, 0.327)
TrnsCondomMax	Maximum rate of condom use in transitory relationships	0.272	(0.265, 0.280)
TrnsCondomMid	Year midpoint of logistic scale-up of condom use in transitory relationships	1997.694	(1997.583, 1997.870)
TrnsCondomRate	Rate of logistic scale-up of condom use in transitory relationships	1.884	(1.811, 1.922)
TrnsFormRate	Transitory relationship formation rate	0.00141	(0.00136, 0.00143)

† We utilized previously published version of EMOD-HIV and recalibrated with most recent data [1, 3]. Modifications to the prior parameterization include PrEP implementation and HIV test sensitivity. A full description of all parameters and references available is at: https://docs.idmod.org/projects/emod-hiv/en/latest/parameter-overview.html and elsewhere[1, 3]

Table S6. Circumcision status quo by county, age group, and year [¥]

	Homa Bay			Kisii			Kisumu			Migori			Nyamira			Siaya		
Year	10-14	15-24	25-49	10-14	15-24	25-49	10-14	15-24	25-49	10-14	15-24	25-49	10-14	15-24	25-49	10-14	15-24	25-49
Pre-2008	0.249	0.249	0.249	0.948	0.948	0.948	0.322	0.322	0.322	0.410	0.410	0.410	0.965	0.965	0.965	0.252	0.252	0.252
2008	0.118	0.235	0.275	0.948	0.948	0.948	0.152	0.303	0.333	0.194	0.385	0.451	0.965	0.965	0.965	0.119	0.243	0.270
2009	0.122	0.240	0.283	0.948	0.948	0.948	0.178	0.315	0.339	0.199	0.388	0.463	0.965	0.965	0.965	0.127	0.250	0.277
2010	0.166	0.285	0.299	0.948	0.948	0.948	0.324	0.389	0.362	0.214	0.395	0.478	0.965	0.965	0.965	0.222	0.310	0.293
2011	0.226	0.355	0.313	0.948	0.948	0.948	0.450	0.479	0.385	0.280	0.423	0.486	0.965	0.965	0.965	0.293	0.375	0.304
2012	0.337	0.486	0.339	0.948	0.948	0.948	0.516	0.559	0.409	0.516	0.530	0.509	0.965	0.965	0.965	0.417	0.483	0.324
2013	0.368	0.565	0.361	0.948	0.948	0.948	0.564	0.634	0.436	0.587	0.614	0.528	0.965	0.965	0.965	0.435	0.549	0.340
2014	0.471	0.710	0.399	0.948	0.948	0.948	0.620	0.712	0.467	0.717	0.726	0.554	0.965	0.965	0.965	0.537	0.659	0.368
2015	0.506	0.811	0.438	0.948	0.948	0.948	0.672	0.790	0.502	0.742	0.813	0.580	0.965	0.965	0.965	0.562	0.739	0.395
2016	0.476	0.894	0.488	0.948	0.948	0.948	0.756	0.844	0.538	0.697	0.891	0.613	0.965	0.965	0.965	0.628	0.841	0.425
2017	0.489	0.937	0.537	0.948	0.948	0.948	0.863	0.889	0.572	0.680	0.949	0.648	0.965	0.965	0.965	0.759	0.917	0.457
2018	0.589	0.925	0.637	0.948	0.948	0.948	0.944	0.925	0.651	0.775	0.976	0.744	0.965	0.965	0.965	0.844	0.959	0.562
2019	0.741	0.904	0.678	0.948	0.948	0.948	0.784	0.943	0.677	0.785	0.976	0.775	0.965	0.965	0.965	0.790	0.976	0.596
2020	0.802	0.901	0.717	0.948	0.948	0.948	0.787	0.925	0.703	0.800	0.976	0.808	0.965	0.965	0.965	0.800	0.976	0.633
2021	0.802	0.909	0.749	0.948	0.948	0.948	0.836	0.913	0.727	0.800	0.976	0.836	0.965	0.965	0.965	0.800	0.976	0.668
2022	0.802	0.917	0.779	0.948	0.948	0.948	0.804	0.947	0.750	0.800	0.976	0.865	0.965	0.965	0.965	0.800	0.976	0.704
2023	0.802	0.926	0.807	0.948	0.948	0.948	0.804	0.947	0.773	0.800	0.976	0.892	0.965	0.965	0.965	0.800	0.976	0.738
2024	0.802	0.935	0.833	0.948	0.948	0.948	0.804	0.947	0.795	0.800	0.976	0.918	0.965	0.965	0.965	0.800	0.976	0.772
2025	0.802	0.944	0.858	0.948	0.948	0.948	0.804	0.947	0.815	0.800	0.976	0.942	0.965	0.965	0.965	0.800	0.976	0.803
2026	0.802	0.949	0.879	0.948	0.948	0.948	0.801	0.947	0.832	0.800	0.976	0.961	0.965	0.965	0.965	0.800	0.976	0.832
2027	0.802	0.953	0.898	0.948	0.948	0.948	0.801	0.947	0.848	0.800	0.976	0.976	0.965	0.965	0.965	0.800	0.976	0.976
2028	0.802	0.955	0.915	0.948	0.948	0.948	0.801	0.947	0.862	0.800	0.976	0.976	0.965	0.965	0.965	0.800	0.976	0.976
2029	0.802	0.955	0.931	0.948	0.948	0.948	0.801	0.947	0.874	0.800	0.976	0.976	0.965	0.965	0.965	0.800	0.976	0.976
2030	0.802	0.955	0.945	0.948	0.948	0.948	0.801	0.947	0.885	0.800	0.976	0.976	0.965	0.965	0.965	0.800	0.976	0.976
2031	0.802	0.955	0.955	0.948	0.948	0.948	0.802	0.947	0.897	0.800	0.976	0.976	0.965	0.965	0.965	0.800	0.976	0.976
2032	0.802	0.955	0.955	0.948	0.948	0.948	0.802	0.947	0.907	0.800	0.976	0.976	0.965	0.965	0.965	0.800	0.976	0.976
2033	0.802	0.955	0.955	0.948	0.948	0.948	0.802	0.947	0.917	0.800	0.976	0.976	0.965	0.965	0.965	0.800	0.976	0.976
2034	0.802	0.955	0.955	0.948	0.948	0.948	0.802	0.947	0.924	0.800	0.976	0.976	0.965	0.965	0.965	0.800	0.976	0.976
2035	0.802	0.955	0.955	0.948	0.948	0.948	0.802	0.947	0.931	0.800	0.976	0.976	0.965	0.965	0.965	0.800	0.976	0.976

[¥] **Source:** Circumcision prevalence prior to 2008 is obtained from the Kenya Demographic and Health Survey, 2003. Prevalence of circumcision from 2008 onward combines prevalence of traditional male circumcision and voluntary medical male circumcision estimates obtained from the Decision-Makers' Program Planning Toolkit 2.

Female sex worker (FSW) and male client of FSW estimation

The sizes of the FSW and male client populations in each setting were estimated based on Ministry of Health surveillance, FSW enumeration studies, and DHS data. Details of this process have been previously published [7]. Briefly, we utilize primary data on mean and standard deviation age of FSWs and employ a 0 to 5 year delay from sexual debut to onset of female sex work using a Weibull distribution; based on data, we assume that FSWs engage in sex work for a for a mean of 5.4 years (95% CI 2 - 9 years), parameterized using a uniform distribution. Triangulating the number of females engaging in sex work at any one time and the duration of sex work, we estimated the lifetime probability that a female would engage in FSW by country. Similarly, we used DHS data on number of men reporting ever having paid for sex in their lifetime and in the last 12 months to inform estimates of ever being a male client of FSWs by setting.

Table S7: Lifetime probability of becoming a female sex worker or male client of FSW in western Kenya by county

	Homa Bay	Kisii	Kisumu	Migori	Nyamira	Siaya
Prevalence of ever becoming FSW (among females)	0.0434	0.1302	0.14982	0.09936	0.05192	0.09406
Prevalence of ever becoming a male client of FSWs (among males)	0.063	0.2035	0.2341	0.1553	0.0811	0.147

Data sources:

Odek WO, Githuka GN, Avery L, Njoroge PK, Kasonde L, Gorgens M, Kimani J, Gelmon L, Gakii G, Isac S, Faran E, Musyoki H, Maina W, Blanchard JF, Moses S. Estimating the size of the female sex worker population in Kenya to inform HIV prevention programming. PLoS One. 2014 Mar 3;9(3):e89180.

Kenya Demographic and Health Survey. Accessed from: https://dhsprogram.com/pubs/pdf/PR143/PR143.pdf on August 29 2024.

Section 2 | Interventions and Analysis Design

2.1 Cost approach and calculations

Costing Approach

Service delivery model: The cost estimates chosen for this analysis were based on assuming delivery of PrEP in health facilities as is the current model of care. In reality, in order to achieve deeper (i.e., more use among the current people accessing PrEP in health facilities) and wider (e.g., more use among people not currently reached by health facility-delivered PrEP) coverage as was modeled with this analysis (90% coverage), alternative delivery models will need to be considered. Differentiated service delivery (DSD) models take advantage of patient-centered existing service delivery channels to better serve individuals and reduce burden on the healthcare.[13–16] Examples of these models include PrEP delivery through community pharmacies, telehealth or mHealth support, or mobile clinics.[14, 15] In theory, these mechanisms will reach a larger number of people and a greater variety of people who are currently being missed by the existing delivery system. However, the potential impacts of these broad scale-up strategies in terms of both epidemic impacts and costs to the healthcare system remain unknown. As such, we used estimates from the literature for costs of facility-delivered PrEP in our main analysis, and included a wider range in sensitivity analyses to understand the range of plausible ICERs that might be applicable under some of these alternate delivery models.

Perspective of costs: We assumed all costs involved in distributing PrEP and HIV care would be borne by the Kenya Ministry of Health. Future studies are needed to examine other cost-sharing models, including client user fees and incentives provided to distributors. We also conducted a version of the analysis considering costs beyond direct medical costs and such as costs from the household perspective (e.g., informal medical supplies, travel time to/from the clinic, transportation costs) and broader societal opportunity costs of HIV (e.g., absenteeism, premature mortality due to HIV). We consider these calculations from the societal perspective to be illustrative of the broader societal burden of HIV and the benefits of PrEP in averting this burden. However, inclusion of the societal perspective in cost-effectiveness analyses of HIV prevention in sub-Saharan Africa is uncommon in the literature, rendering it difficult to source reliable inputs and compare these findings with other publications. As such, the cost-effectiveness results from the societal perspective are included here as a sensitivity analysis (Section 3).

Cost sources and adjustments: Cost estimates were derived from the published literature. Estimates for some components (e.g., RDT costs) varied widely among published studies, and in these cases, we prioritized studies based on geographic relevance, recency, sample size, and methodology. For all studies conducted prior to 2017, we adjusted for inflation to 2021 $USD using World Bank inflation estimates [17]. For the cost of illness estimates which collected data in South Africa rather than Kenya, we adjusted the estimates by taking a ratio of the GDP per capita of the two countries to approximate the differences in purchasing power parity between the two settings, since PPP or health-specific CPIs were not reliably available in these settings.[18, 19] While PPP-based approaches are more commonly used than GDP per capita, in this case that adjustment approach was producing estimates that were much higher than the expected range (based on other Kenya-based costing studies) due to PPP fluctuations during high periods of inflation and the COVID-19 pandemic. So, though it is less commonly used, we applied a ratio of the GDP per capita of the two countries as our adjustment factor instead.

Table S8. Cost parameter calculations [¥]

Cost Parameter	Estimate (USD)	Year[†]	Data source	Calculation details, notes
Annual health care costs (among those not on ART)				
HIV-positive CD4 < 200	110.30	2021	[20]	Adjusted for inflation and GDP/capita ratio using this approach:<br>Step 1: Adjust South Africa value in 2012 USD for inflation to be in 2021 USD ($374.08 = $167*2.24)<br>● Cost of health care use, CD4 count <200 cells per µL, not in HIV care (per person-year) in South Africa= $167<br>● USD Inflation Rate between time of costing (2012) and 2021: 2.24 =4.7/2.1<br>Step 2: Adjust South Africa 2021 USD value by multiplying by the Kenya GDP/cap ratio ($374.08*0.295)<br>● South Africa 2021 GDP per capita in $USD = 7,055; Kenya $USD = 2,082<br>● Kenya GDP/ ZA GDP ratio adjustment: (2,082/7,055)= 0.295
HIV-positive CD4 200 - 349	30.38	2021	[20]	Adjusted for inflation and GDP/capita ratio (see steps above)
HIV-positive CD4 > 350	8.59	2021	[20]	Adjusted for inflation and GDP/capita ratio (see steps above)
End of life care	105.68	2021	[20]	Adjusted for inflation and GDP/capita ratio (see steps above)
Annual ART provision costs	140.89	2021/ 2016	[21–23]	Includes 20% mark up on ART drug costs to account for supply chain (2021 USD). ART delivery costs are from an in-country micro-costing study and include lab tests and staff encounters (2016 USD).<br>Step 1: Calculate annual ART cost accounting for 20% supply chain cost mark-up ($57.89= $48.24*1.2)<br>● Monthly ART cost (per pack, 30 for Dolutegravir/Lamivudine/Tenofovir 50/300/300mg tablet)= $4.02<br>● Annual ART cost= $48.24= $4.02*12<br>● 20% supply chain cost mark-up= *1.2<br>Step 2: Calculate total cost of ART delivery ($83=45+38) and add to Step 1<br>● $45 lab costs<br>● $38 staff encounters
Cost of 30 days of PrEP medication	7.00	2021/ 2019	[24, 25]	We identified multiple studies containing cost estimates for PrEP medication in Kenya (Roberts 2017, Wanga 2019, Mangale 2022) and chose a value to use for our analysis that was within the range of those study results. For example, the price of 30 days of PrEP drugs in these 3 studies were $6.75 (Roberts), $7.02 (Wanga), and $6.75 (Mangale), so we picked $7.00 as our estimate for this study to be conservative. [24–26]
Cost of PrEP initiation visit	6.50	2021/ 2019	[24, 25]	We identified multiple studies containing cost estimates for PrEP initiation in Kenya (Roberts 2017, Wanga 2019, Mangale 2022) and chose a value to use for our analysis that was within the range of those study results. For example, Roberts et al calculated the

				visit cost for PrEP initiation was $13.28 total, though $6.75 of that was medications, so we used the difference ($6.53) as an estimate for a PrEP initiation visit. The Roberts, Wanga, and Mangale estimates all include personnel, lab testing, and supply costs. To include some of the fixed costs (e.g., facilities, capital, training) would lead to a higher estimate of PrEP initiation, but for the purposes of this study we used the variable costs only, as we were scaling up PrEP to a hypothetical coverage of 90% and the application of those fixed costs to this scenario was not plausible.
Cost of PrEP continuation visit	5.00	2021/ 2019	[24, 25]	We identified multiple studies containing cost estimates for PrEP continuation visits in Kenya (Roberts 2019, Wanga 2021, Mangale 2022) and chose a value to use for our analysis that was within the range of those study results, following a similar logic to estimating costs of PrEP initiation as described above.
Facility-based HIV-positive test	3.68	2017	[12]	Data from micro-costing study. Inputs include screening test kit, other supply costs, and personnel costs.
Facility-based HIV-negative test	2.64	2017	[12]	Data from micro-costing study. Inputs include screening test kit, other supply costs, and personnel costs.
HIV RDT for PrEP initiation	2.00	2022/ 2022	[24, 27, 28]	We identified multiple studies and supplier reports containing cost estimates for HIV RDTs for PrEP initiation in Kenya, as well as internal expert opinion.
Referral if the HIV test for PrEP initiation is positive	4.00	2016/ 2022	[12, 26]	The sum of $0.31 provider time for referral if positive test (Mangale), $3.68 for an HIV test (Meisner).

¥ PrEP: pre-exposure prophylaxis, ART: antiretroviral therapy. All HIV test, ART and PrEP provision costs include personnel, overhead, and supplies.
† Inflation adjustment using data from the World Bank (% inflation, consumer prices) (40) applied only if reference provides cost estimates for years prior to 2016.

2.2 Intervention design

Summary of Implementation Scenarios

Table S9. Implementation scenario definitions

Scenario	Description
**SDCs***	Serodiscordant couples; individuals who are aware their partner has HIV and is not on ART
**Higher-risk AGYW**	Adolescent girls and young women who have 2 concurrent partners partnerships (of any kind) simultaneously
**All AGYW**	All adolescent girls and young women age 15-24
**Higher-risk women**	Women aged 25-49 who have had 2 or more partners in the past 3 months
**Higher-risk ABYM**	Adolescent boys and young men who have 2 partnerships (of any kind) simultaneously
**All ABYM**	All adolescent boys and young men age 15-24
**Higher-risk men**	Men aged 25-49 who have had 2 or more partners in the past 3 months

*We assume 50% of individuals with diagnosed HIV in a partnership disclose their status to their partners.

HIV testing approach

Background facility-based HIV testing is incorporated into the model as part of the HIV care cascade. Individuals undergo HIV testing at rates based on empiric data on testing coverage in the past 12 months from Kenya. In addition to the background facility-based HIV testing, we also distributed HIV testing to individuals as part of PrEP implementation (below). Individuals who are not known to be HIV-positive and have at least one sexual partner can receive HIV testing and subsequent PrEP provision upon testing HIV-negative. HIV testing is provided every 3 months thereafter to individuals seeking to refill PrEP in line with WHO guidelines for PrEP provision.

We assumed the test used was a provider-administered Ab rapid diagnostic test (RDT). Test sensitivity increased from 0.0% in the first 19 days of infection to 99.9% after 120 days of infection. These values were derived from a review of empiric data in the literature, including data from *Taylor et al* on the cumulative probability of a negative test result for a third- & fourth-generation HIV test at various time points in an HIV-positive individual.[8] We first calculated the inverse of cumulative probability to obtain the cumulative probability of a positive test result. To adjust for RDTs (as these were excluded from Taylor et al. 2015), we shifted the inverse cumulative probability 10 days forward. Finally, we made an adjustment to Ab RDT at day 45 by using mean of day 30 and 60 (0.865 vs. 0.950). See Cox, Wu et al 2024 [9] for additional information on test sensitivity parameterization in this model.

Table S10. Assumed RDT sensitivity

**Day from exposure:**	0-18.9	19-21.9	22-29.9	30-44.9	45-59.9	60-89.9	90-119.9	120+
**Ab RDT sensitivity:**	00.0%	20.0%	37.0%	78.0%	86.5%	95.0%	97.0%	99.9%

PrEP provision

Briefly, the care pathway for PrEP modeled in this analysis was assumed to follow the steps observed in clinical trials. First, providers screen interested clients for HIV risk and PrEP contraindications.[10, 11] Providers then counsel eligible clients regarding PrEP use and safety. Next, clients complete HIV testing to confirm negative status, assuming the sensitivity of a standard provider-administered RDT. Those who test HIV-negative are dispensed PrEP and those testing HIV-positive are referred to health care clinics for confirmatory testing and treatment. Providers dispense a 1-month PrEP supply at initiation and a 3-month supply thereafter. At subsequent visits, clients completed a self-administered risk and PrEP safety assessment and HIV testing. Those who are eligible can obtain a PrEP refill. We assumed 75% of

eligible individuals would continue taking PrEP at the end of their supply. At each 1-month timestep in the model, the age and partnership status of each individual is re-evaluated, and our pre-determined proportion (90% for the main analysis) of those individuals begin this pathway to initiate PrEP. In this way, we are modeling a PrEP uptake among a set of individuals based on risk behavior which varies in the model, rather than a pre-determined constant PrEP coverage over several years. This process is depicted in **Figure S3** and key parameters are highlighted in **Table S10** below. We include costs for all aspects of the care pathway in the microcosting that informed our cost inputs for each PrEP scenario.[12]

Figure S3. Schematic depicting model implementation of PrEP delivery

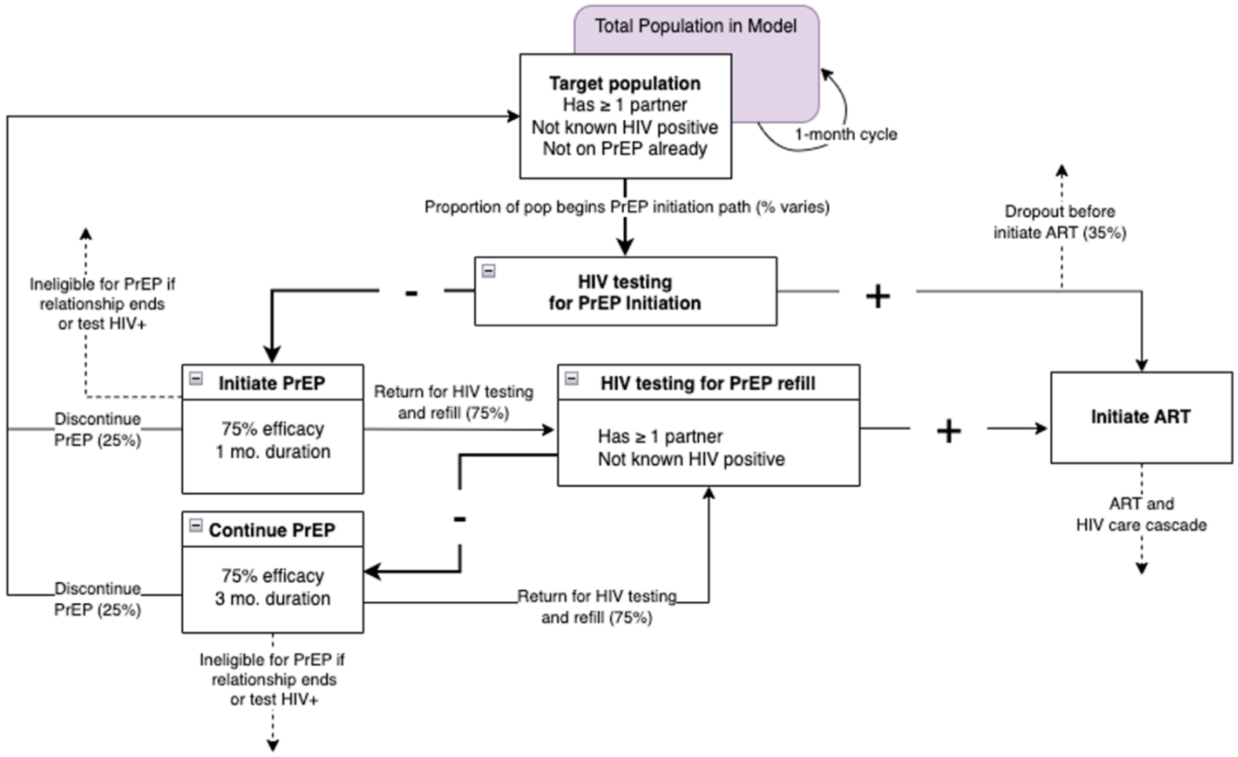

Table S11. Summary of key PrEP implementation assumptions

Parameter	Implementation assumption
PrEP efficacy	75%
Duration on PrEP at initiation	1 month
Duration on PrEP at continuation	3 months
Likelihood of discontinuation at end of current PrEP prescription if still eligible	25%
Proportion initiating PrEP	90% of all individuals eligible, as defined by subgroups in each evaluation scenario

Section 3 | Results and Sensitivity Analyses

3.1 Additional main analysis results

Table S12. PrEP outcomes by scenario in western Kenya (Median, 90% CI)*

	Counterfactual HIV incidence in priority subgroup with no PrEP availability++ (2022 – 2027)	Mean proportion of population in subgroup (age 15-65) (2022-2027)	PrEP Coverage in population age 15-65 (2022-2027)	Infections Averted (2022-2027)	Deaths Averted (2022-2042)	DALYs Averted (2022-2042)	Incremental Costs ($ Millions) (2022-2042)	ICER† (2022-2042)
Baseline	**N.A.**	**N.A.**	**0%**	(ref)	(ref)	(ref)	(ref)	(ref)
SDCs	**Men: 4.338 (0.822) Women: 10.634 (0.636)**	**0.41%** (0.37 – 0.44%)	**0.37%** (0.34 - 0.4%)	**17.61%** (15.58 - 20.07%)	**4.13%** (2.83 - 5.49%)	**32,517** (17,668 – 46,856)	**$8** ($7 - 9)	**$245** ($179 - $435)
Higher-risk AGYW	**1.151 (0.102)**	**2.26%** (2.16 – 2.37%)	**2.03%** (1.94 - 2.13%)	**14.93%** (12.17 - 16.95%)	**0.93%** (-0.42 - 2.5%)	**12,069** (-627 – 29,211)	**$61** (58 - 64)	**$4,745** ($2,059 - $22,515)
Broad AGYW	**0.472 (0.035)**	**9.20%** (9.01 – 9.48%)	**8.28%** (8.11 - 8.53%)	**21.29%** (19.21 - 23.59%)	**1.26%** (-0.24 - 2.79%)	**20,501** (6,907 – 36,625)	**$256** ($250 - 263)	**$12,351** ($7,050 - $33,955)
Higher-risk Women	**1.751 (0.132)**	**1.18%** (1.07 – 1.24%)	**1.06%** (0.96 - 1.12%)	**10.3%** (7.99 - 13.05%)	**1.67%** (0.28 - 3.23%)	**16,090** (-850 – 29,976)	**$31** ($28 - 33)	**$1,898** ($1,002 - $6,771)
Higher-risk ABYM	**0.287 (0.033)**	**2.06%** (1.92 – 2.19%)	**1.85%** (1.73 - 1.97%)	**4.52%** (2.33 - 7.17%)	**0.32%** (-1.04 - 1.51%)	**5,539** (-8,099 – 21,333)	**$57** ($53 - 60)	**$6,622** ($2,447 - $35,253)
Broad ABYM	**0.184 (0.018)**	**5.23%** (5.00 – 5.41%)	**4.71%** (4.5 - 4.87%)	**6.68%** (4.05 - 8.44%)	**0.54%** (-0.74 - 1.77%)	**6,055** (-7,525 – 22,622)	**$147** ($140 - 151)	**$15,945** ($6,358 - $151,949)
Higher-risk Men	**0.458 (0.032)**	**4.68%** (4.53 – 4.82%)	**4.21%** (4.08 - 4.34%)	**21.58%** (20.03 - 24.02%)	**6.73%** (5.27 - 8.04%)	**53,026** (35,331 – 67,706)	**$124** ($120 - 128)	**$2,351** ($1,831 - $3,494)

*Health impacts are compared to baseline scenario of no PrEP availability.

†Dominated model runs of each scenario were excluded from ICER calculations: SDCs: 0; Higher-risk AGYW: 6; Broad AGYW: 1; Higher-risk Women: 6; Higher-risk ABYM: 27; Broad ABYM: 18; Higher-risk Men: 0.

++Counterfactual HIV incidence per 100 person-years in the scenario's priority population had there been no PrEP provision; mean (sd)

Figure S4. PrEP initiations by scenario

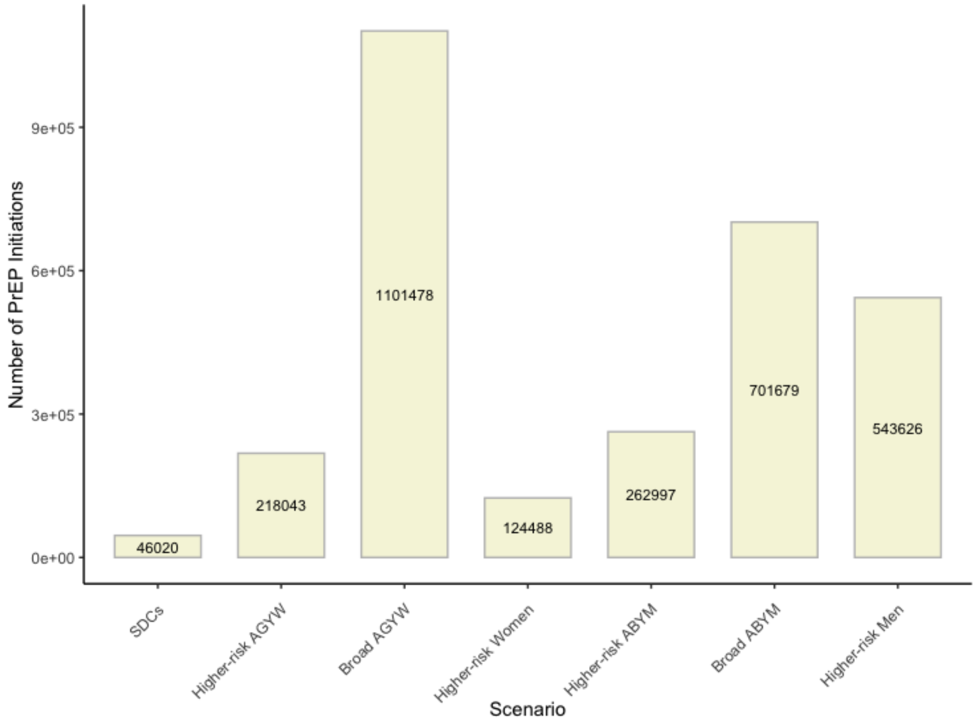

Figure S5. HIV Incidence Among Ages 18-49 (2020 - 2027)

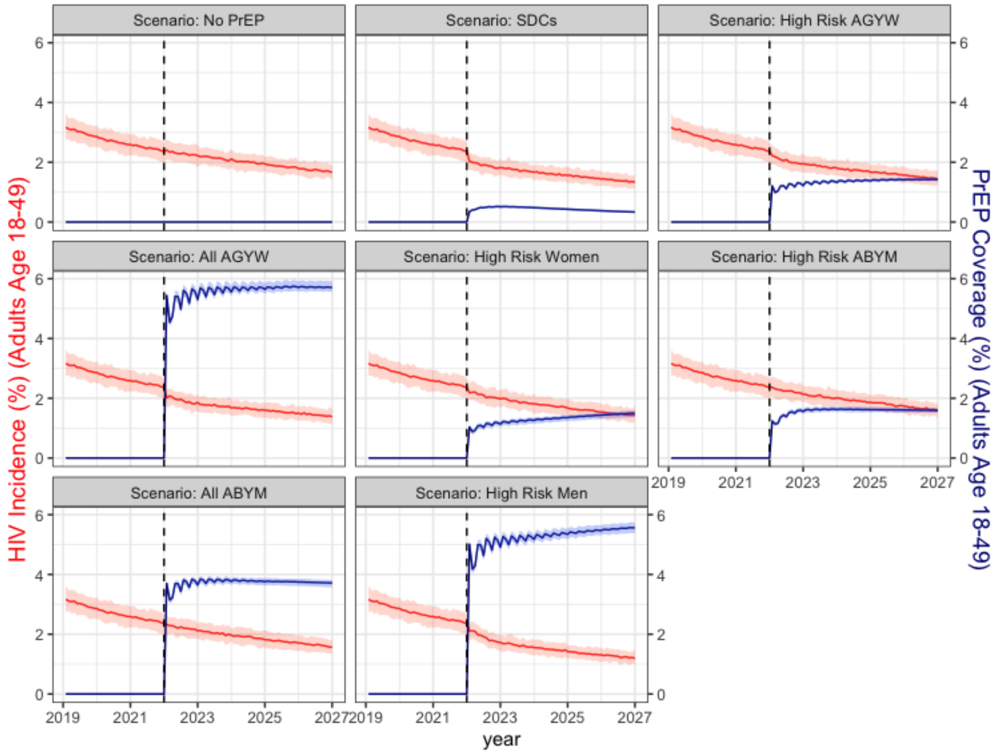

The red lines depict HIV incidence, which has a visible drop-off upon commencement of the modeled PrEP implementation in most scenarios. The blue line depicts the proportion of adults aged 18-49 who are on PrEP at a given time. In the first few iterations of this cycle, there are oscillations in PrEP coverage which eventually smooth out as the eligible population moves through the PrEP testing, initiation, and continuation cycles. In the first month time-step, 90% of the eligible individuals initiate PrEP, then in the next time-step 75% of the eligible people continue for 3 months of PrEP while the remainder discontinue and return to the main population pool. At the next timestep, everyone is re-evaluated again and more are initiated on PrEP (up to 90% of eligible people), and so on. Thus eventually the timing of the PrEP continuation cycles is less uniform among the whole population, which causes the 3-month oscillations seen in the first year of PrEP implementation.

Table S13. Proportion of scenarios that are cost-effective by county

**County**	**SDCs**	**Higher-risk AGYW**	**Broad AGYW**	**Higher-risk Women**	**Higher-risk ABYM**	**Broad ABYM**	**Higher-risk Men**
**Homa Bay (20%)**	99.0%	2.1%	0.0%	64.4%	2.4%	0.0%	42.0%
**Siaya (16%)**	95.9%	1.3%	0.0%	35.9%	1.9%	0.0%	4.0%
**Kisumu (14%)**	88.7%	1.3%	0.0%	38.8%	0.0%	0.0%	0.0%
**Migori (13%)**	94.8%	1.4%	0.0%	46.2%	0.0%	0.0%	1.0%
**Nyamira (3%)**	93.5%	24.0%	0.0%	29.5%	5.9%	0.0%	0.0%
**Kisii (3%)**	97.1%	0.0%	0.0%	1.7%	0.0%	0.0%	0.0%

Note: Cost-effectiveness threshold used was $1000/DALY averted, and includes scenarios that are cost-saving. Dominated runs were removed.

Figure S6. Cost-effectiveness plane of main analysis results

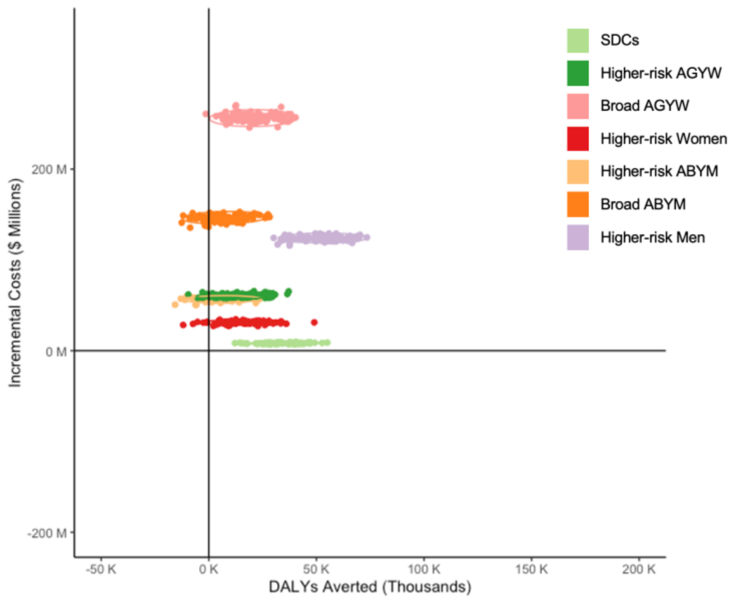

Incremental outcomes versus baseline from 2022 – 2042 with all outcomes discounted at 3%
Note: scenarios with negative DALYs averted were due to stochastic variation in these scenarios.

Figure S7a. Components of 20-year total cost relative to baseline by category in each scenario

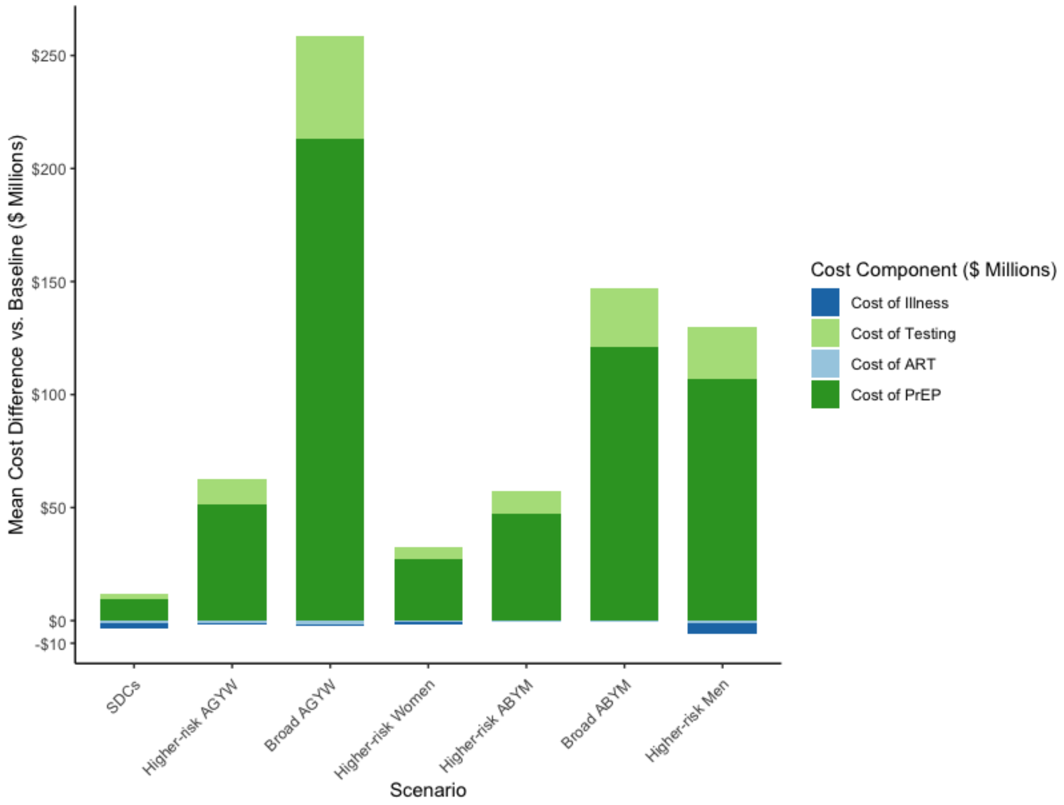

Figure S7b. Total 20-year discounted costs by category in each scenario

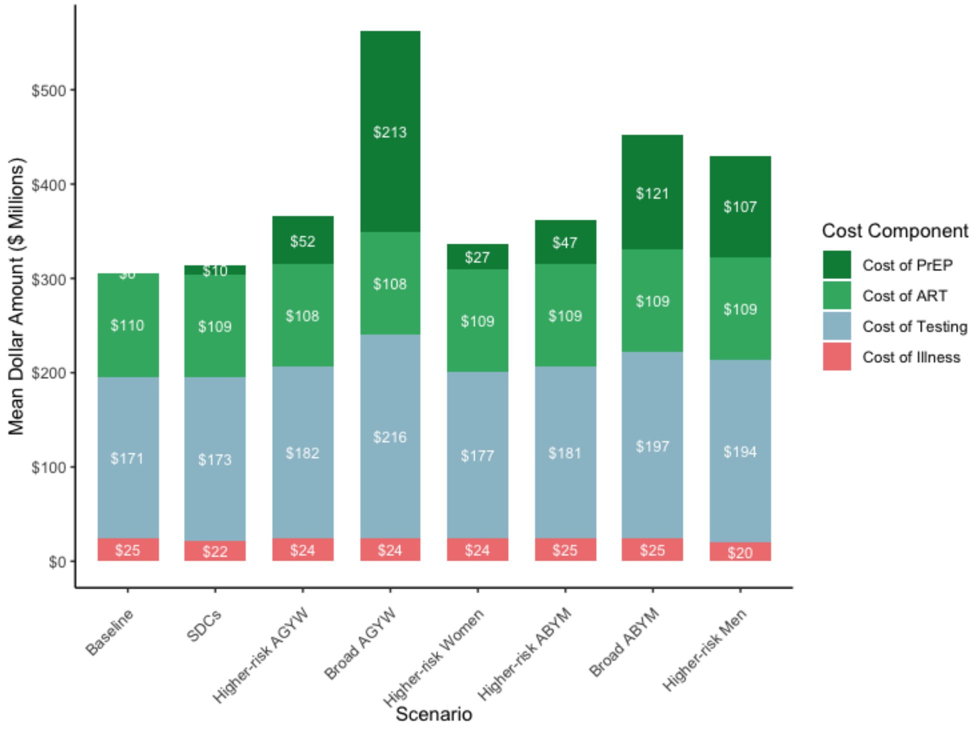

Figure S7c. Proportions of total 20-year discounted costs by category in each scenario

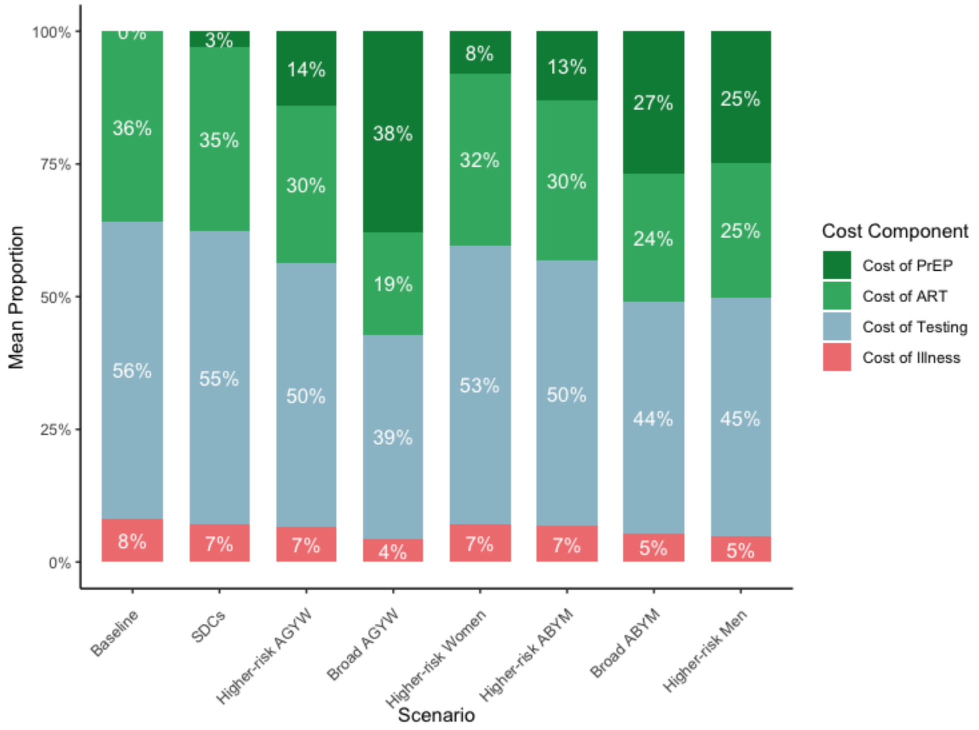

Table S14a. Total 20-year discounted costs by category in each scenario

Scenario	Cost of HIV-related Illness	Cost of Testing	Cost of ART	Cost of PrEP
**Baseline**	$24,853,938.06	$170,990,169.61	$109,751,115.47	$ -
**SDCs**	$22,280,425.98	$173,305,218.14	$108,707,095.74	$ 9,506,734.75
**Higher-risk AGYW**	$24,440,882.84	$181,839,959.91	$108,491,989.11	$ 51,582,894.17
**Broad AGYW**	$24,228,637.95	$216,486,404.98	$108,075,852.02	$213,284,578.05
**Higher-risk Women**	$23,887,128.87	$176,749,197.21	$109,106,912.54	$ 26,919,664.04
**Higher-risk ABYM**	$24,705,115.66	$181,157,066.01	$109,313,872.73	$ 47,067,229.98
**Broad ABYM**	$24,625,191.85	$197,200,911.34	$109,158,476.44	$120,950,795.56
**Higher-risk Men**	$20,354,285.27	$193,935,266.37	$108,515,233.94	$106,818,842.43

Table S14b. Incremental 20-year discounted costs by category in each scenario versus baseline

Scenario	Cost of HIV-related Illness	Cost of Testing	Cost of ART	Cost of PrEP
**Baseline**	(ref)	(ref)	(ref)	(ref)
**SDCs**	$(2,573,512.08)	$ 2,315,048.53	$(1,044,019.73)	$ 9,506,734.75
**Higher-risk AGYW**	$ (413,055.22)	$10,849,790.31	$(1,259,126.35)	$ 51,582,894.17
**Broad AGYW**	$ (625,300.11)	$45,496,235.38	$(1,675,263.45)	$213,284,578.05
**Higher-risk Women**	$ (966,809.19)	$ 5,759,027.60	$ (644,202.93)	$ 26,919,664.04
**Higher-risk ABYM**	$ (148,822.40)	$10,166,896.40	$ (437,242.74)	$ 47,067,229.98
**Broad ABYM**	$ (228,746.21)	$26,210,741.74	$ (592,639.03)	$120,950,795.56
**Higher-risk Men**	$(4,499,652.79)	$22,945,096.77	$(1,235,881.53)	$106,818,842.43

Figure S8a. Total 5-year undiscounted costs by category in each scenario

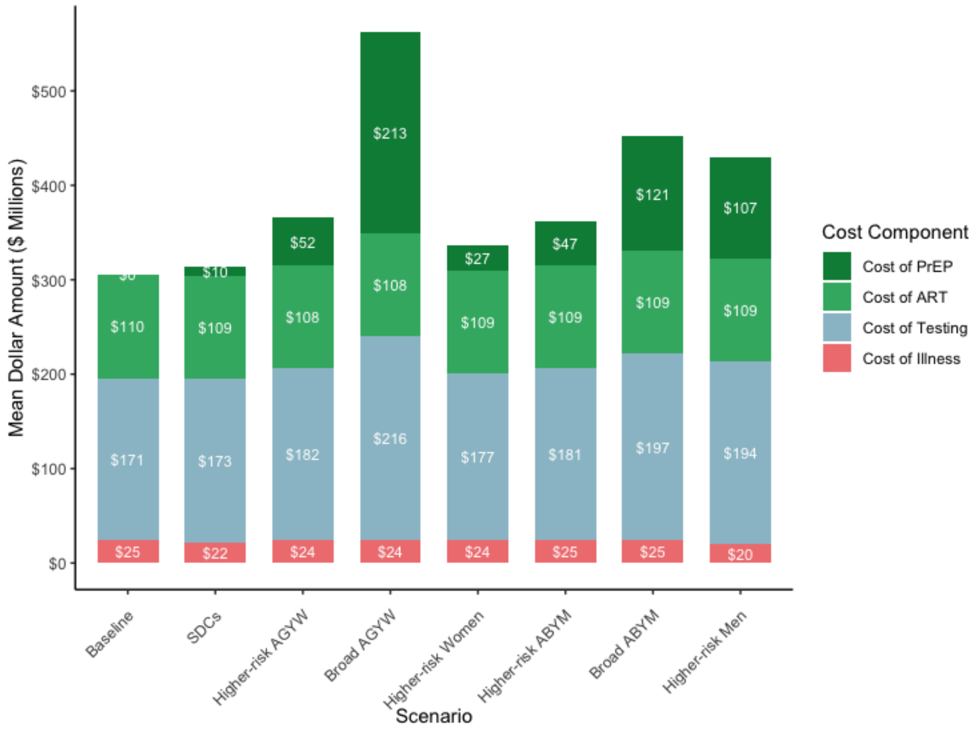

Figure S8b. Proportions of 5-year undiscounted costs by category in each scenario

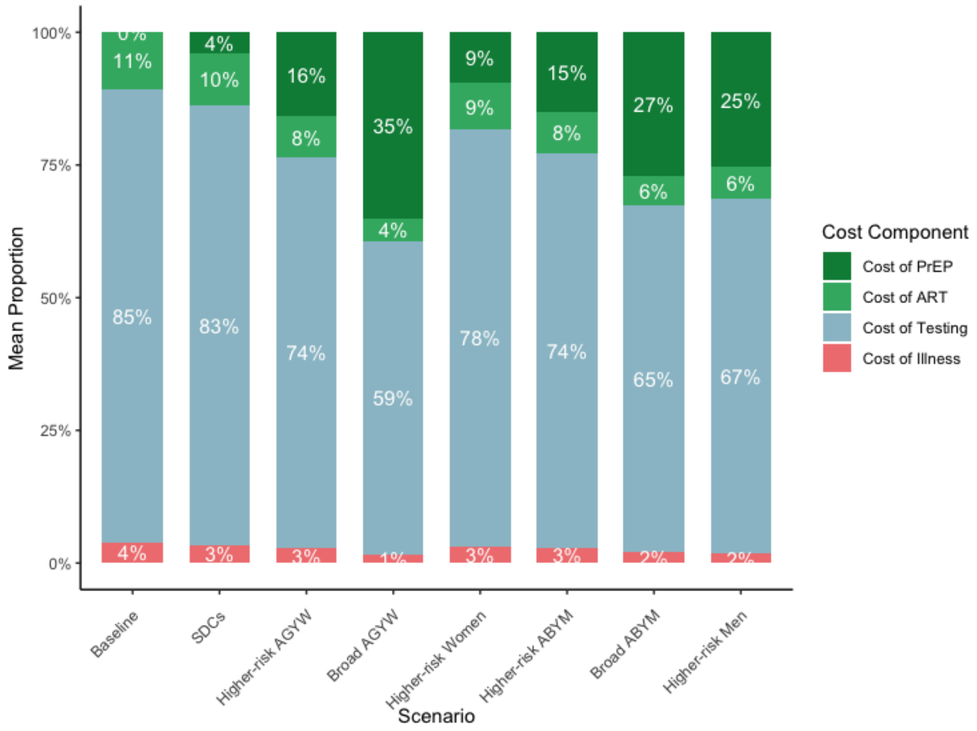

3.2 Sensitivity Analyses

We conducted several one-way sensitivity analyses to understand the impact of changes in parameter values on the results of our main analysis. We first did one-way analysis using the upper and lower bound of several key parameter estimates including PrEP efficacy, DALY weights, PrEP costs, and test costs. These upper and lower bounds were selected based on the range of estimates found in the literature review to parameterize our main analysis. The ICERs resulting from each of these changes are depicted in the tornado diagram in the main report and included in the tables below. We expect the relative impact of these parameter changes to be similar across scenarios, and so here we report only the ICER impact on one scenario: high-risk AGYW.

We also explored three additional scenarios to contextualize our findings: the inclusion of some background use of oral PrEP in the model, a coverage of 50% of each eligible group using PrEP rather than 90%, and taking the societal perspective in calculating the cost-effectiveness.

Table S15. Summary of sensitivity analyses

Parameter	Main Analysis	Low Estimate	High Estimate
**One-way sensitivity analyses**			
PrEP Efficacy	75%	60%	90%
DALY weights<br>HIV-positive and on ART<br>HIV+ (CD4 >350)<br>HIV+ (CD4 200-350)<br>HIV+ (CD4 <200)	Estimate:<br>0.078<br>0.274<br>0.312<br>0.642	Lower bound of 95% CI:<br>0.052<br>0.184<br>0.217<br>0.470	Upper bound of 95% CI:<br>0.111<br>0.377<br>0.418<br>0.792
PrEP Drug Cost	$7.00	5	15
PrEP Initiation Cost	$6.50	5	15
PrEP Cont. Cost	$5.00	3	10
HIV Test Cost	$2.00	0.50	8
Referral	$4.00	1	8
**Additional sensitivity analyses evaluated**			
1. Coverage	90%	50%	*NA*
2. Cost perspective	MOH perspective	Societal perspective	*NA*

Table S16. ICER results of one-way sensitivity analyses

Variable	Level	Value	ICER
**PrEP Efficacy**	Low	60%	5,855
	High	90%	3,946
DALY weights	Low	Lower Bound	5,909
	High	Upper Bound	3,682
HIV Test Cost	Low	$0.50	4,477
	High	$8	5,817
Positive Referral	Low	$1	4,744
	High	$8	4,746
PrEP Init. Visit Cost	Low	$5	4,672
	High	$15	5,161
PrEP Cont. Visit Cost	Low	$3	4,486
	High	$10	5,392
Monthly PrEP Drug Cost	Low	$5	3,872
	High	$15	8,241

Sensitivity Analysis 1: Lower PrEP Coverage
This scenario assumed 50% PrEP coverage in each subgroup vs 90% coverage in the main analysis.

Table S17. PrEP outcomes by scenario (Median, 90% CI) for Lower PrEP Coverage*

	**PrEP Coverage (age 15-65) (2022-2027)**	**Infections Averted (2022-2027)**	**Deaths Averted (2022-2042)**	**DALYs Averted (2022-2042)**	**Incremental Costs ($ Millions) (2022-2042)**	**ICER† (2022-2042)**
Baseline	**0%**	(ref)	(ref)	(ref)	(ref)	(ref)
SDCs	**0.2%** (0.18 - 0.21%)	**10.08%** (6.82 - 13.67%)	**2.58%** (1 - 4.77%)	**22504** (-427 – 39,588)	**$4** ($3 - 5)	**$229** ($97 - $548)
Higher-risk AGYW	**1.19%** (1.13 - 1.25%)	**8.62%** (6.15 - 12.55%)	**0.77%** (-1.21 - 2.54%)	**8217** (-14,961 – 25,583)	**$35** (34 - 37)	**$12,282** ($1,354 - $30,869)
Broad AGYW	**4.85%** (4.74 - 5%)	**13.86%** (10.25 - 16.67%)	**0.98%** (-1.39 - 2.76%)	**11613** (-12739 – 33,454)	**$149** ($146 - 154)	**$20,549** ($4,358 - $73,154)
Higher-risk Women	**0.6%** (0.56 - 0.64%)	**6.01%** (2.73 - 10.14%)	**1.28%** (-0.52 - 3.01%)	**10796** (-9,871 – 27,496)	**$18** ($16 - 19)	**$17,416** ($612 - $17,121)
Higher-risk ABYM	**1.09%** (1.04 - 1.15%)	**2.67%** (-0.71 - 6.31%)	**0.3%** (-1.57 - 2.44%)	**393** (-15,610 – 20,789)	**$34** ($32 - 36)	**$13,979** ($1,477 - $78,330)
Broad ABYM	**2.78%** (2.67 - 2.85%)	**4.45%** (1.16 - 8.26%)	**0.64%** (-1.19 - 2.32%)	**6746** (-16,516 – 23,070)	**$86** ($83 - 89)	**$17,421** ($3,374 - $69,959)
Men with 2+ partners	**2.46%** (2.39 - 2.53%)	**15.63%** (12.27 - 19.45%)	**5.79%** (4.05 - 7.73%)	**45187** (15,156 – 69,765)	**$71** ($69 - 74)	**$2,105** ($1,027 - $4,762)

*Health impacts are compared to baseline scenario of no PrEP availability.
†Dominated model runs of each scenario were excluded from ICER calculations: SDCs: 6; Higher-risk AGYW: 16; Broad AGYW: 14; Higher-risk women: 10; Higher-risk ABYM: 33; Broad ABYM: 22; Higher-risk Men: 0.

Table S18. ICERs by scenario and geography for Lower PrEP Coverage

**County (Baseline HIV Prevalence)**	**SDCs**	**Higher-risk AGYW**	**Broad AGYW**	**Higher-risk Women**	**Higher-risk ABYM**	**Broad ABYM**	**Higher-risk Men**
**Homa Bay (20%)**	$113	$1,378	$3,750	$667	$1,218	$3,330	$778
**Siaya (16%)**	$152	$1,049	$3,831	$567	$2,545	$2,805	$1,104
**Kisumu (14%)**	$163	$1,762	$4,846	$635	$1,586	$4,049	$1,221
**Migori (13%)**	$148	$1,123	$6,931	$557	$1,409	$5,828	$1,243
**Nyamira (3%)**	$41	$807	$7,268	$453	$1,216	$4,453	$1,603
**Kisii (3%)**	$80	$1,461	$9,786	$1,189	$1,561	$4,680	$3,123

Sensitivity Analysis 2: Societal perspective of costs

Table S19. Societal perspective costs and calculations

**Category**	**Estimate (Range)**	**References and Notes**
**Household Costs**		
Transportation costs to care location	$1.96 ($1.00 - $4.00)	Katana 2020 [29]; Brennan 2019[30]; Simoni 2021 [31]
Travel time for patient per visit	15 minutes (10 - 90 minutes)	Ortblad 2021[28]; Katana 2020[29]; Wong 2020 [32]
Wait time for patient in care setting per visit	15 minutes (0 - 30 minutes)	Roche 2021 [15]; Ortblad 2021 [10]
Average annual income (prorated to value lost wages from travel and wait time)	$1,729 annually	World Bank. [33] Assumed 40h/work/week, 50 weeks/year. Assumed 3% increase per year.
Lost wages for PLWH or their caregivers	2.7 lost days per month	Katana 2020 [29]
Costs of informal care and ancillary expenses	$2.34 ($0 - $3.70) per month	Katana 2020 [29]
**Societal Opportunity Costs**		
Economic productivity loss due to absenteeism		Calculated as product of below inputs, as instructed in Mensah 2020. [34]
% productive time lost per year from HIV: Absenteeism	6% of days, or 1.8 days per month	Katana 2020. HIV causes individuals to lose 6% of working days due to absenteeism [29]
% productive time lost per year from HIV: Presenteeism	3% of days, or 0.9 days per month	Katana 2020. HIV causes individuals to lose 3% of working days to presenteeism [29]
GDP per worker	$10,681 annually	World Bank. [33] Assumed future growth of 5% annually. [34]
Labor force participation rate	73%	World Bank [33]
Employment rate	94.3%	World Bank [33]
Economic productivity loss due to premature death		Calculated as product of below inputs, as instructed in Mensah 2020. [34]
GDP per worker	$10,681 (2021 USD)	World Bank. [33] Assumed future growth of 5% annually. [34]
Labor force participation rate	73%	World Bank [33]
Employment rate	94.3%	World Bank [33]

From the full societal perspective, all scenarios were considered cost-saving. This was largely driven by the reduction in premature mortality due to HIV. **Figure S9** depicts the cost-effectiveness plane for each scenario.

Figure S9. Cost-effectiveness plane of societal perspective results

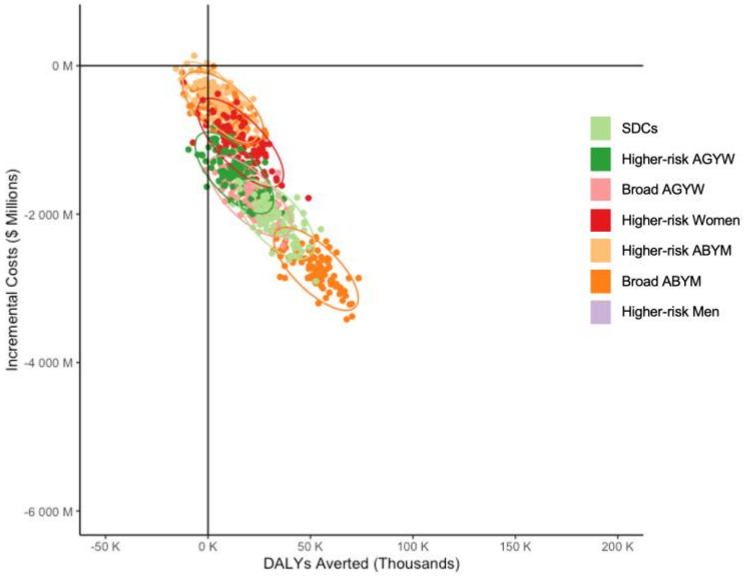

A key limitation of this sensitivity analysis is that few CEA studies in Kenya have been done with the societal perspective, despite being best practice per the Second Panel on Cost-Effectiveness reference case. Articles identified by the authors during a preliminary literature search found that cost-of-illness studies are nearly always limited to direct medical costs or MOH perspective, with a few exceptions which incorporate household costs such as informal medical care or patient waiting time, and almost no studies which include societal costs such as absentee- or presenteeism. As such, limited evidence informed the estimates in this analysis, and there are limited options to compare results to other studies for validity.

Supplemental References

1. Bershteyn A, Klein D (2015) STI and HIV Model Introduction. IAS
2. Welcome to EMOD HIV modeling — HIV Model documentation. https://docs.idmod.org/projects/emod-hiv/en/latest/. Accessed 2 Sep 2024
3. Relationships and contact networks — HIV Model documentation. In: Institute for Disease Modeling. https://docs.idmod.org/projects/emod-hiv/en/latest/sti-model-relationships.html. Accessed 28 Aug 2024
4. Klein DJ, Bershteyn A, Eckhoff PA (2014) Dropout and re-enrollment: Implications for epidemiological projections of treatment programs. AIDS. https://doi.org/10.1097/QAD.0000000000000081
5. Bershteyn A, Klein DJ, Wenger E, Eckhoff PA, Bershteyn * A, Klein DJ Description of the EMOD-HIV Model v0.7.
6. Raftery AE, Bao L (2010) Estimating and Projecting Trends in HIV/AIDS Generalized Epidemics Using Incremental Mixture Importance Sampling. Biometrics 66:1162–1173
7. Bershteyn A, Mutai KK, Akullian AN, Klein DJ, Jewell BL, Mwalili SM (2018) The influence of mobility among high-risk populations on HIV transmission in Western Kenya. Infect Dis Model 3:97
8. Taylor D, Durigon M, Davis H, et al (2014) Probability of a false-negative HIV antibody test result during the window period: a tool for pre- and post-test counselling. http://dx.doi.org/101177/0956462414542987 26:215–224
9. Cox SN, Wu L, Wittenauer R, et al (2024) Impact of HIV self-testing for oral pre-exposure prophylaxis scale-up on drug resistance and HIV outcomes in western Kenya: a modelling study. Lancet HIV. https://doi.org/10.1016/S2352-3018(23)00268-0
10. Ngure K, Ortblad KF, Bardon AR, et al (2022) Efficiency of 6-month PrEP dispensing with HIV self-testing in Kenya: an open-label, randomised, non-inferiority, implementation trial. Articles Lancet HIV. https://doi.org/https://doi.org/10.1016/S2352-3018(22)00126-6
11. Masyuko S, Mukui I, Njathi O, et al (2018) PREP ROLL OUT IN A NATIONAL PUBLIC SECTOR PROGRAM: THE KENYAN CASE STUDY. Sex Health 15:578
12. Meisner J, Roberts DA, Rodriguez P, et al (2021) Optimizing HIV retesting during pregnancy and postpartum in four countries: a cost-effectiveness analysis. J Int AIDS Soc 24:e25686
13. NASCOP;, Kenya Ministry of Health Kenya AIDS Strategic Framework II (2020/1 - 2024/5).
14. Vanhamel J, Rotsaert A, Reyniers T, Nöstlinger C, Laga M, Van Landeghem E, Vuylsteke B (2020) The current landscape of pre-exposure prophylaxis service delivery models for HIV prevention: a scoping review. BMC Health Serv Res 20:1–18
15. Roche SD, Wairimu N, Mogere P, Kamolloh K, Odoyo J, Kwena ZA, Bukusi EA, Ngure K, Baeten JM, Ortblad KF (2021) Acceptability and Feasibility of Pharmacy-Based Delivery of Pre-Exposure Prophylaxis in Kenya: A Qualitative Study of Client and Provider Perspectives. AIDS Behav 25:3871–3882
16. Ortblad KF, Mogere P, Bukusi E, Ngure K, Baeten JM (2020) Pharmacy delivery to expand the reach of PrEP in Africa. J Int AIDS Soc. https://doi.org/10.1002/jia2.25619
17. Turner HC, Lauer JA, Tran BX, Teerawattananon Y, Jit M (2019) Adjusting for Inflation and Currency Changes Within Health Economic Studies. Value in Health 22:1026–1032

18. Edejer T-TT, Baltussen R, Adam T, Hutubessy R., Acharya A, Evans DB, Murray CJL (2003) Making choices in health: WHO guide to cost-effectiveness analysis. Geneva
19. World Health Organization (2019) WHO Guide on Standardization of Economic Evaluations of Immunization Programmes.
20. Eaton JW, Menzies NA, Stover J, et al (2014) Health benefits, costs, and cost-effectiveness of earlier eligibility for adult antiretroviral therapy and expanded treatment coverage: A combined analysis of 12 mathematical models. Lancet Glob Health 2:e23–e34
21. Phillips AN, Cambiano V, Johnson L, et al (2021) Potential Impact and Cost-Effectiveness of Condomless-Sex-Concentrated PrEP in KwaZulu-Natal Accounting for Drug Resistance. J Infect Dis 223:1345–1355
22. Larson BA, Bii M, Halim N, Rohr JK, Sugut W, Sawe F (2018) Incremental treatment costs for HIV-infected women initiating antiretroviral therapy during pregnancy: A 24-month micro-costing cohort study for a maternal and child health clinic in Kenya. PLoS One 13:e0200199
23. The Global Fund (2023) Pooled Procurement Mechanism Reference Pricing: ARVs.
24. Wanga V, Peebles K, Obiero A, et al (2021) Cost of pre-exposure prophylaxis delivery in family planning clinics to prevent HIV acquisition among adolescent girls and young women in Kisumu, Kenya. PLoS One 16:e0249625
25. Roberts DA, Barnabas R V., Abuna F, et al (2019) The role of costing in the introduction and scale-up of HIV pre-exposure prophylaxis: evidence from integrating PrEP into routine maternal and child health and family planning clinics in western Kenya. J Int AIDS Soc 22:e25296
26. Mangale D OKHJMPKCMN (2022) Comparing the cost of six-month PrEP dispensing with interim HIV self-testing to the standard-of-care three-month PrEP dispensing with clinic-based testing in Kenya. AIDS Annual Meeting (Abstract #EPE327)
27. Peebles K, Mugwanya KK, Irungu E, et al (2021) Low costs and opportunities for efficiency: a cost analysis of the first year of programmatic PrEP delivery in Kenya's public sector. BMC Health Serv Res 21:1–8
28. Kuo AP, Ekwunife O, Mogere P, et al (2023) Costs of providing pharmacy-initiated PrEP in Kenya: findings from a pilot study. Abstracts From CROI 2023 Conference on Retroviruses and Opportunistic Infections CROI
29. Katana P V., Abubakar A, Nyongesa MK, Ssewanyana D, Mwangi P, Newton CR, Jemutai J (2020) Economic burden and mental health of primary caregivers of perinatally HIV infected adolescents from Kilifi, Kenya. BMC Public Health 20:1–9
30. Brennan AT, Maskew M, Larson BA, Tsikhutsu I, Bii M, Vezi L, Fox MP, Venter WDF, Ehrenkranz P, Rosen S (2019) Who is seeking antiretroviral treatment for HIV now? Characteristics of patients presenting in Kenya and South Africa in 2017-2018. J Int AIDS Soc 22:e25358
31. Simoni JM, Beima-Sofie K, Wanje G, Mohamed ZH, Tapia K, McClelland RS, Ho RJY, Collier AC, Graham SM (2021) "Lighten This Burden of Ours": Acceptability and Preferences Regarding Injectable Antiretroviral Treatment Among Adults and Youth Living With HIV in Coastal Kenya. J Int Assoc Provid AIDS Care. https://doi.org/10.1177/23259582211000517/ASSET/IMAGES/LARGE/10.1177_23259582211000517-FIG1.JPEG

32. Wong KLM, Brady OJ, Campbell OMR, Banke-Thomas A, Benova L (2020) Too poor or too far? Partitioning the variability of hospital-based childbirth by poverty and travel time in Kenya, Malawi, Nigeria and Tanzania. Int J Equity Health 19:1–15
33. World Bank Open Data | Data. https://data.worldbank.org/. Accessed 14 Dec 2023
34. Mensah J, Korir J, Nugent R, Hutchinson B (2020) Combating Noncommunicable Diseases in Kenya An Investment Case. Washington DC
